# OTUD1 promotes pathological cardiac remodeling and heart failure by targeting STAT3 in cardiomyocytes

**DOI:** 10.7150/thno.83340

**Published:** 2023-04-17

**Authors:** Mengyang Wang, Xue Han, Tianxiang Yu, Minxiu Wang, Wu Luo, Chunpeng Zou, Xiuyun Li, Gao Li, Gaojun Wu, Yi Wang, Guang Liang

**Affiliations:** 1 Department of Pharmacy and Institute of Inflammation, Zhejiang Provincial People's Hospital, Affiliated People's Hospital, Hangzhou Medical College, Hangzhou, Zhejiang, 310014, China.; 2 Chemical Biology Research Center, School of Pharmaceutical Sciences, Wenzhou Medical University, Wenzhou, Zhejiang, 325035, China.; 3 Key Laboratory of Natural Medicines of the Changbai Mountain, Ministry of Education, Yanbian University, Yanji 133002, China.; 4 Department of Cardiology, the First Affiliated Hospital of Wenzhou Medical University, Wenzhou, Zhejiang, 325035, China.; 5 Department of Ultrasonography, the Second Affiliated Hospital, Wenzhou Medical University, Wenzhou, Zhejiang, 325000, China.

**Keywords:** OTUD1, Angiotensin II, Deubiquitination enzyme, STAT3, Heart failure

## Abstract

**Rationale:** Understanding the molecular mechanisms of deleterious cardiac remodeling is important for the development of treatments for heart failure. Recent studies have highlighted a role of deubiquitinating enzymes in cardiac pathophysiology. In the present study, we screened for alteration of deubiquitinating enzymes in experimental models of cardiac remodeling, which indicated a potential role of OTU Domain-Containing Protein 1 (OTUD1).

**Methods:** Wide-type or OTUD1 knockout mice with chronic angiotensin II infusion and transverse aortic constriction (TAC) were utilized to develop cardiac remodeling and heart failure. We also overexpressed OTUD1 in mouse heart with AAV9 vector to validate the function of OTUD1. LC-MS/MS analysis combined with Co-IP was used to identify the interacting proteins and substrates of OTUD1.

**Results:** We found that OTUD1 is elevated in mouse heart tissues following chronic angiotensin II administration. OTUD1 knockout mice were significantly protected against angiotensin II-induced cardiac dysfunction, hypertrophy, fibrosis and inflammatory response. Similar results were obtained in the TAC model. Mechanistically, OTUD1 bounds to the SH2 domain of STAT3 and causes deubiquitination of STAT3. Cysteine at position 320 of OTUD1 exerts K63 deubiquitination to promote STAT3 phosphorylation and nuclear translocation, thereby increasing STAT3 activity to induce inflammatory responses, fibrosis, and hypertrophy in cardiomyocytes. Finally, OTUD1 overexpression by AAV9 vector increases Ang II-induced cardiac remodeling in mice and OTUD1-regulated responses can be inhibited by blocking STAT3.

**Conclusion:** Cardiomyocyte OTUD1 promotes pathological cardiac remodeling and dysfunction by deubiquitinating STAT3. These studies have highlighted a novel role of OTUD1 in hypertensive heart failure and identified STAT3 as a target of OTUD1 in mediating these actions.

## Introduction

The prevalence of hypertension is rising globally. More than 30% of the adult population is affected by hypertension, representing a major risk factor for stroke, myocardial infarction, and heart failure 1. Left ventricular workload is chronically elevated in hypertensive patients, leading to left ventricular remodeling, impaired relaxation, and an increased risk of heart failure 2. The renin-angiotensin system (RAS) has been identified as a key mediator of hypertensive cardiac remodeling characterized by ventricular hypertrophy, cardiac inflammation and fibrosis 3. However, RAS blockers targeting are not able to completely reverse cardiac remodeling and heart failure 4. Other treatment modalities also have limitations. Therefore, identifying regulatory molecules involved in heart failure and elucidating their mechanisms of action carries significant clinical value.

Most chronic pathological conditions involve the altered levels of regulatory/causative cellular proteins. In eukaryotic cells, two main systems regulate protein turnover, and these include the ubiquitin-proteasome system and lysosomal systems 5, 6. The proteasomal system consists of the proteasome, ubiquitin (Ub), the ubiquitination machinery, and deubiquitinases (DUBs). This system is involved in a range of cell signal transductions, cell fate determination, inflammatory responses, and other important cellular/protein life activities 7, 8. It is not surprising that a dysfunction of ubiquitinating enzymes is associated with cancer, and cardiovascular and neurodegenerative diseases 9-12. Likewise, altered deubiquitinating enzymes are associated with cardiovascular diseases, infectious diseases, and cancer 13-16. These studies highlight the importance of ubiquitinating and deubiquitinating factors as an important mechanism underlying diseases.

Recent studies have shown that genes involved in proteasome-mediated ubiquitin-dependent protein catabolic process are differentially expressed in hypertension 17. Ubiquitin, E1-3, and proteasomes regulate cardiac homeostasis and dysfunction 18. However, detailed screening of deubiquitinating factors and evidence of downstream functional significance is not currently known. In the present study, we examined the expression profile of DUBs in the angiotensin II-challenged mouse model of hypertensive cardiac dysfunction. From this transcriptomic study, we identified significantly upregulated level of a DUB, OTU Domain-Containing Protein 1 (OTUD1, also known as DUBA7), in heart tissues of hypertensive mice. Using OTUD1 knockout mice, we demonstrate that angiotensin II (Ang II)-mediated cardiac hypertrophy, fibrosis, and functional deficits are prevented when OTUD1 is deficient. This activity of OTUD1 was not limited to Ang II as transaortic constriction model showed the same results. Our detailed mechanistic studies showed that OTUD1 interacts with signal transducer and activator of transcription 3 (STAT3) and leads to deubiquitination and increased phosphorylation of STAT3. These studies identify a deubiquitinating enzyme, OTUD1, as an important regulator in hypertensive cardiac disease and reveal STAT3 as its downstream substrate.

## Methods

### Reagents

Angiotensin II (cat# 4474913) was purchased from Aladdin (Shanghai, China). Small interfering RNA against OTUD1 and scrambled sequences were purchased from Genepharma (Shanghai, China). Flag-OTUD1, His-STAT3, HA-Ub, HA-K48, and HA-K63 plasmids, and AAV9 for OTUD1 expression and an empty vector were obtained from Genechem (Shanghai, China). Antibody against OTUD1 (orb185712) was purchased from Biorbyt (Cambridge, UK). Antibodies against cardiac myosin heavy chain (MyHC; ab50967), transforming growth factor-β1 (TGF-β1; ab179695), collagen 1 (COL-1; ab34710), Lamin B (ab133741), alpha actinin (ab68167), and vimentin (ab8978) were obtained from Abcam (Cambridge, UK). Antibodies against STAT3 (12640S), phospho (Tyr705)-STAT3 (9145s), and GAPDH (5174) were purchased from Cell Signaling Technology (MA, USA). Rabbit IgG (B900610) and antibodies against His (6005-1-lg), Flag (20543-1-AP) and HA (51064-2-AP) were obtained from Proteintech (IL, USA).

Angiotensin II ELISA kit was purchased from Shanghai Tongwei Biological Technology Co., Ltd (Shanghai, China). Atrial natriuretic peptide (ANP) and creatine kinase-MB (CKMB) kits (cat# H180, E006-1-1) were purchased from Nanjing Jiancheng Bioengineering Institute (Nanjing, China). FITC-conjugated wheat-germ agglutinin (WGA-FTIC; cat# GTX01502) was purchased from Gene Tex (CA, USA). Rhodamine-conjugated Phalloidin (Phalloidin-Rho; cat# CA1610-300T), slide mounting media with DAPI (cat# S2110), hematoxylin and eosin (H&E) kit (cat# G1120), Picro Sirius Red stain (cat# S8060) and Masson's Trichrome kit (cat# G1340) were purchased from Solarbio Life Sciences (Beijing, China). Stattic (CAS No. 19983-44-9), a STAT3 inhibitor 19, was purchased from MedChemExpress (New Jersey, USA).

### Collection of human heart samples

Hypertrophic myocardium samples were collected from heart failure patients who underwent pacemaker electrode replacement. Control samples were obtained from arrhythmia patients without heart failure who underwent pacemaker electrode replacement. All the experiments involving human samples were approved by the Ethics Committee of The First Affiliated Hospital of Wenzhou Medical University (Wenzhou, China; approval no. KY2022-156), and conformed to the principles outlined in the Declaration of Helsinki.

### Mouse model of cardiac hypertrophy and remodeling

Wildtype C57BL/6J mice were obtained from the Animal Center of Wenzhou Medical University. OTUD1^-/-^ mice on a C57BL/6 background were provided by Dr. You Fuping (Peking University Health Science Center, Beijing, China) 20. Primer sequences for genotyping are provided in Table S1. Mice were maintained in a specific-pathogen-free environment and given food and water ad lib. All animal care and experimental procedures were approved by the Wenzhou Medical University Animal Policy and Welfare Committee (Approval Document No. wydw 2021-1016). All animals received humane care according to the National Institutes of Health guidelines (USA). Animals were housed with a 12:12 h light-dark cycle at a constant room temperature and fed a standard rodent diet. The animals were acclimatized to the laboratory for at least 2 weeks before initiating the studies. All animal experiments were performed and analysed by blinded experimenters. Treatment groups were assigned in a randomised fashion.

(1) To induce cardiac hypertrophy and excessive remodeling, we used chronic Ang II infusion and transverse aortic constriction (TAC). For the Ang II model, male wildtype and OTUD1^-/-^ mice aged 6-8 weeks were infused with Ang II (1 μg/kg/min) or saline through an Alzet micro-osmotic pump (Model 1004; CA, USA) for four weeks. The osmotic pump was subcutaneously implanted on the back of each mouse. Blood pressure and body weights were measured weekly, and mice were sacrificed at week 4 to collect serum and heart tissues.

(2) For the TAC model, mice were anesthetized with isoflurane. Hair was removed and an incision was made to expose the trachea. A breathing tube was inserted through the mouth into the trachea and connected to the ventilator. The second rib was cut along the sternum, and a thoracotomy device was placed to prop the thoracic rib. After separation of the thymus, the upper margin of the aortic arch and the left common carotid artery were fully exposed. A 6-0 suture was passed under the aortic arch with a hook, followed by the ligation of a 27 G needle with the separated aortic arch along the direction of the aorta. Finally, the needle was removed to constrict the ascending aorta. Mice were sacrificed at week 4 to collect serum and heart tissues. Blood samples were used to prepare serum and measure the levels of ANP, CK-MB and Ang II by ELISA.

For echocardiography, mice were anesthetized with isoflurane one day before killed. Cardiac short-axis section indices and E/A ratio were obtained using the multi-mode small animal ultrasound imaging system (Vevo 3100; FUJIFILM Visual Sonics, Canada).

(3) We also expressed OTUD1 in C57BL/6J mice to examine cardiac hypertrophy and remolding. To do this, WT mice were infected with adeno-associated virus serotype 9 (AAV9) encoding OTUD1 (AAV9-OTUD1) or an empty vector (AAV9-NC). Mouse OTUD1 gene was cloned into AAV-9 by Genechem Co. LTD (Shanghai, China). Six-week-old mice were injected with AAV9 through tail vein (2x10^11^ particles/mouse/month) for 4 weeks. Mice were then infused with saline or Ang II through an Alzet micro-osmotic pump (Model 1004; CA, USA) for 2 weeks. An experimental subgroup also included STAT3 inhibitor Stattic-treated mice. Stattic was administered at 10 mg/kg/d for the final two weeks. A total of 30 wildtype mice were used for this model. Experimental groups included (n = 6 per group): AAV9-NC, AAV9-OTUD1, AAV9-NC +Ang II, AAV9-OTUD1+Ang II, and AAV9-OTUD1+Ang II + Stattic (10 mg/kg).

### Immunostaining and histological analysis

Heart tissues from mice were processed for both frozen sectioning and paraffin embedding. Paraffin-embedded tissues were sectioned into 5 μm thick slices for histological analyses. Tissue sections were stained with hematoxylin and eosin (H&E), Masson's Trichrome stain, and Picro Sirius Red. To examine hypertrophy, frozen heart tissues were sectioned at 5 μm thickness, permeabilized with 0.1% Triton X-100 for 10 min, and then blocked with 5% bovine serum albumin for 30 min. Sections were stained with FITC-WGA (1:200) for 30 min at 37 °C. Images were obtained using an epifluorescence Nikon microscope (Nikon, Tokyo, Japan).

For immunofluorescence staining, 5 μm thick tissue sections were permeabilized with 0.1% Triton X-100 for 10 min and blocked with 2% bovine serum albumin for 45 min. Samples were then incubated with primary antibodies at 4 °C overnight. Antibodies included OTUD1 (1:200), α-actinin (1:100), and vimentin (1:200). Fluorophore-conjugated secondary antibodies were used for detection. Slides were counterstained with DAPI for 5 min. Images were taken using the Nikon A1 laser confocal microscope (Nikon, Japan).

### Culture, transfection, and staining of cells

HEK-293T cells (cat# SCSP-502) were obtained from the Stem Cell Bank of the Chinese Academy of Sciences (Shanghai, China). HEK-293T cells were cultured in DMEM medium containing 4.5 g/L glucose, 10% fetal bovine serum, and 1% penicillin/streptomycin. Cells were transfected with siRNA or expression plasmids using Lipofectamine 3000 (L3000-015; Thermo Fisher, Massachusetts, USA).

To isolate primary cardiomyocytes (neonatal rat ventricular myocytes; NRVMs), a previously published protocol was used 21. Briefly, neonatal rats were sacrificed. Heart tissues were removed, washed in PBS, and digested using a mixture of pancreatin and collagenase. Fibroblasts were removed by differential adherence culture. Fibroblasts were cultured in DMEM medium containing 4.5 g/L glucose and 10% fetal bovine serum. Myocytes were also cultured in the same media with the addition of 5-BrdU (HY-15910, Med Chem Express, New Jersey, USA). After 48 h of culture, NVRMs exhibited regular pulsation and were used for experiments. For some experiments, a rat cardiomyocyte-like cell line H9c2, obtained from the Shanghai Institute of Biochemistry and Cell Biology, was used.

Primary mouse peritoneal macrophages (MPMs) were prepared from C57BL/6 wildtype mice. Mice were administered 1 ml of 4% thioglycolate in PBS intraperitoneally. Three days later, peritoneal cells were collected and incubated in DMEM/F12 supplemented with 10% fetal bovine serum for 4 h. Cells were then incubated at 37 °C for 6 h and washed with PBS to remove the non-adherent cells. The remaining adherent cells were used as the peritoneal macrophages described in the experiments.

NVRMs were seeded on glass-bottom dishes. Following various treatments, cells were fixed with 4% formaldehyde at room temperature for ten minutes. Cells were permeabilized with 0.5% Triton X-100 solution for five minutes. Cells were then stained with fluorophore conjugated Phalloidin (CA1610; Beijing Solarbio Science & Technology Co., Ltd, Beijing, China) at room temperature in the dark for thirty minutes. Finally, cells were counterstained with DAPI (C0065; Beijing Solarbio Science & Technology), and imaged.

### Silencing of OTUD1 gene

Gene silencing in cells was achieved using specific siRNA sequences. OTUD1 siRNAs were purchased from Gene Pharma Co. Ltd. (Shanghai, China). Custom siRNAs were synthesized for rat OTUD1. The sequences of OTUD1 siRNA and scrambled siRNA are shown in Table S2. siRNA at a final concentration of 50 nM was mixed with 2 μL Lipofectamine2000 (Thermo Fisher, CA, USA) in 200 μL serum-free Opti-MEM and incubated at room temperature for 20 min. The mixture was added to neonatal rat ventricular myocytes cells in DMEM containing fetal bovine serum but lacking antibiotics, followed by incubation for 24 h.

### Western blotting and co-immunoprecipitation analysis

Total proteins from mouse tissues and cell culture samples were extracted with RIPA buffer (P0013C; Beyotime Biotechnology, Shanghai, China). Protein levels were measured by the Bradford assay (Thermo Fisher) and an equal amount of protein was loaded and separated by sodium dodecyl sulfate-polyacrylamide gel electrophoresis (SDS-PAGE). Samples were transferred to PVDF membranes and blocked with 5% skim milk for one hour at room temperature. Primary antibody incubations were carried out overnight at 4 ℃. Membranes were then washed, and horseradish peroxidase-conjugated secondary antibodies were applied for 1 h at room temperature. Immunore-activity was detected by chemiluminescence (ECL) reagent.

For co-immunoprecipitation studies, a small amount of lysate was removed as input. The remaining samples were incubated with target antibodies at 4 ℃ overnight. Subsequently, magnetic beads were added to the protein lysate and incubated at 4 ℃ for 2 h. The supernatant was discarded after centrifugation. The magnetic bead mixture was washed with PBS and buffer containing SDS was added. The samples were used for subsequent western blot experiments.

### RNA-sequencing analysis

Total RNA was isolated from heart tissues of wildtype mice infused with saline or Ang II for 4 weeks, using TRIzol reagent (cat# 15596018; Thermo Fisher). RNA integrity was assessed by electrophoresis with denaturing agarose gel. Poly(A) RNA was fragmented into small pieces and reverse transcribed to cDNA by SuperScript II Reverse Transcriptase (cat# 1896649; Thermo Fisher). After pretreatments, the ligated products were amplified with PCR. The average size for the final cDNA library was 300 ± 50 bp. Finally, 2x150 bp paired-end sequencing (PE150) was performed on an Illumina Novaseq 6000 (LC-BioTechnology Co., Ltd., Hangzhou, China). The differentially expressed genes (DEGs) were selected with fold change >2 or fold change <0.5 and *P*-value < 0.05. Gene-set enrichment analysis was performed as described by LC-Bio (https://www.lc-bio.cn/).

### Quantitative Real-time PCR analysis

Total RNA was isolated using TRIzol and reverse-transcribed to cDNA using PrimeScript RT reagent Kit (cat# DRR037A; Takara, Japan). Quantitative polymerase chain reaction (PCR) was carried out using TB Green Premix Ex Taq II (cat# RR82WR; Takara, Japan) in the CFX96 Touch Real-Time PCR Detection System (Bio-Rad; Hercules, CA, USA). Data was normalized to *Actb*. Primers were purchased from Sangon Biotech (Shanghai, China) (Table S1).

### LC-MS/MS analysis

OTUD1 antibody was added to NVRM lysates for IP. Rabbit IgG was used as a negative control. Then, the LC-MS/MS analysis was carried out by PTM Bio Co., Ltd (Zhejiang, China). Finally, we screened out the substrate proteins that could bind to OTUD1 according to the score and the mass of detected proteins.

### Statistical analysis

Results from the experiments were reported as Mean ± SEM. To identify differences, we used one-way analysis of variance (ANOVA) with the LSD correction for multiple comparisons. For samples that required for a single mouse to be measured more than once, data sets were analyzed independently using two-way repeated-measures ANOVA analysis with a single pooled variance and a Tukey correction for pairwise comparisons within groups for each data set. A statistically significant difference was obtained at *p* < 0.05. All data analyses were implemented in SPSS 21.0.

## Results

### OTUD1 is upregulated in heart tissues of mice challenged with Ang II

**Figure 1:**
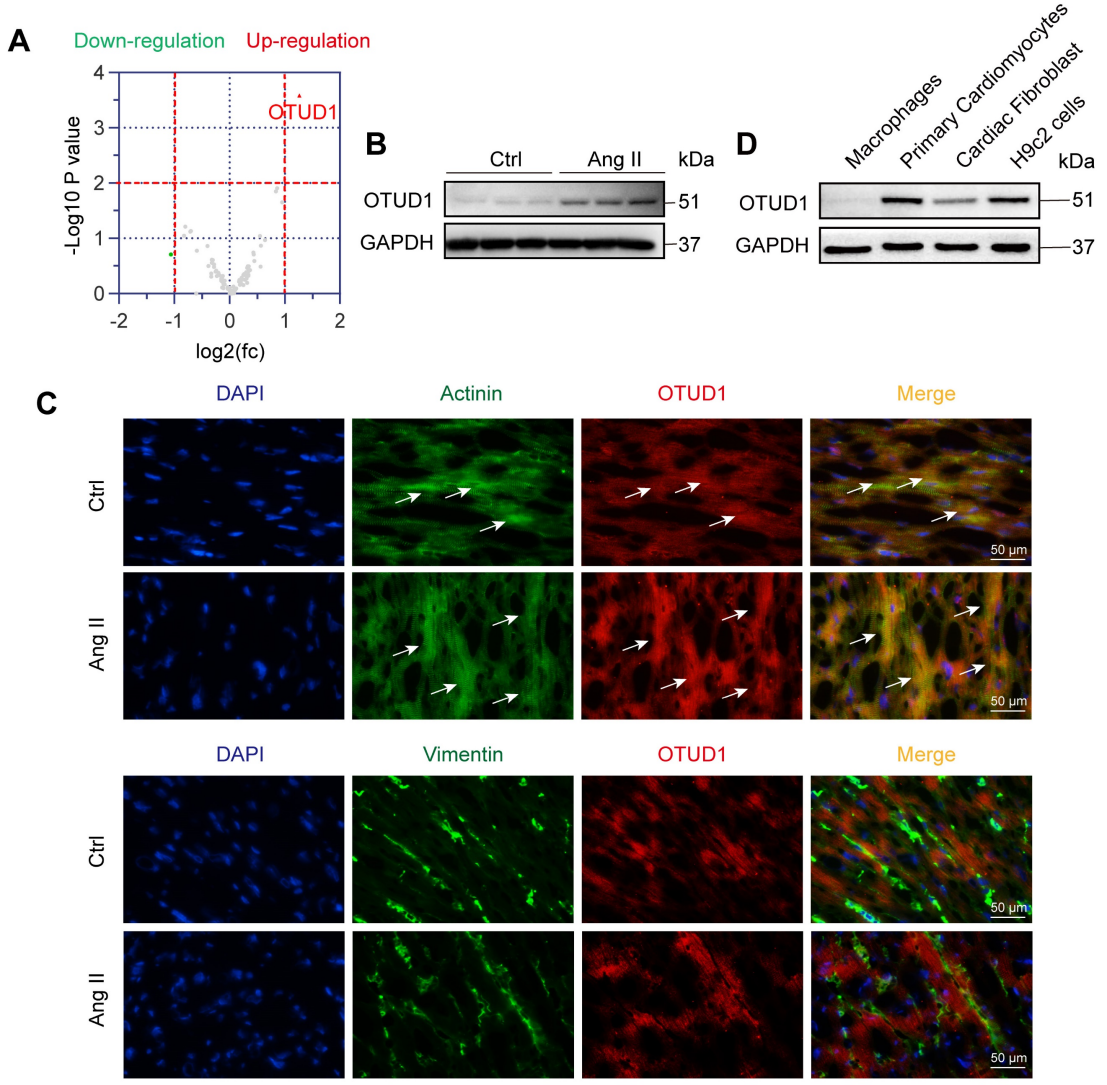
OTUD1 is induced in Ang II-mediated cardiac hypertrophy and fibrosis model. **(A)** RNA sequencing was performed in heart tissues of C57BL/6 mice infused with saline (Ctrl) or Ang II for 4 weeks. Expression profile of deubiquitylating enzymes (DUBs) showing up- (red) and down-regulated (green) genes. Grey points represent DUBs showing no statistical difference compared to Ctrl. Fold change ˃ 2 times and *p* ˂ 0.05 indicate statistically significant differences. **(B)** Representative western blot analysis for OTUD1 levels in Ctrl and Ang II-infused heart tissues. GAPDH was used as loading control. **(C)** Immunofluorescence staining of mouse heart tissues for OTUD1 (red), vimentin (green), and sarcomeric alpha actinin (green). Sections were counterstained with DAPI (blue). Arrows showing OTUD1-positive cells [scale bar = 50 μm]. **(D)** Representative western blot analysis for OTUD1 protein levels in macrophages, primary cardiomyocytes (neonatal ventricular myocytes), cardiac fibroblast and H9c2 cells. GAPDH was used as loading control.

We first wanted to profile altered DUBs in the setting of cardiac hypertrophy and remodeling. For this, we used the well-established mouse model of chronic infusion of Ang II at 1 μg/kg/min. RNA-sequencing from heart tissues of mice administered with or without Ang II for 4 weeks revealed altered expression of DUBs (Figure 1A, Figure S1A). In this profile, we noted significantly increased levels of OTUD1 in heart tissues of Ang II-infused mice. We then confirmed the increased levels of OTUD1 in heart tissue lysates of Ang II-infused mice using immunoblotting (Figure 1B, Figure S1B). We examined the mRNA expression of OTUD1 in heart tissues from human subjects. Compared with the non-hypertrophic myocardium tissues, the mRNA level of OTUD1 was up-regulated in hypertrophic myocardium tissues of heart failure patients (Figure S1C). This changing profile of OTUD1 is consistent with our findings in mice. To identify the source of increased OTUD1 in the heart, we stained the tissues for OTUD1, α-actinin (marker of cardiomyocytes), and vimentin (marker of fibroblasts). Our data shows that the likely source of increased OTUD1 in heart tissues following Ang II administration are the cardiomyocytes (Figure 1C). Specifically, OTUD1 immunoreactivity was noted in cells expressing actinin but not vimentin. We then confirmed these findings in isolated cellular fractions consisting of primary macrophages from wildtype mice, cardiomyocytes (neonatal rat ventricular myocytes), and neonatal rat cardiac fibroblasts. Immunoblotting showed robust levels of OTUD1 in primary cardiomyocytes and H9c2 cardiomyocyte-like cells (Figure 1D, Figure S1D). These results suggest that OTUD1 levels are increased following Ang II challenge in mice and that OTUD1 potentially relates to cardiac hypertrophy and remodeling.

### OTUD1 deficiency protects against Ang II-induced myocardial hypertrophy and fibrosis

**Figure 2:**
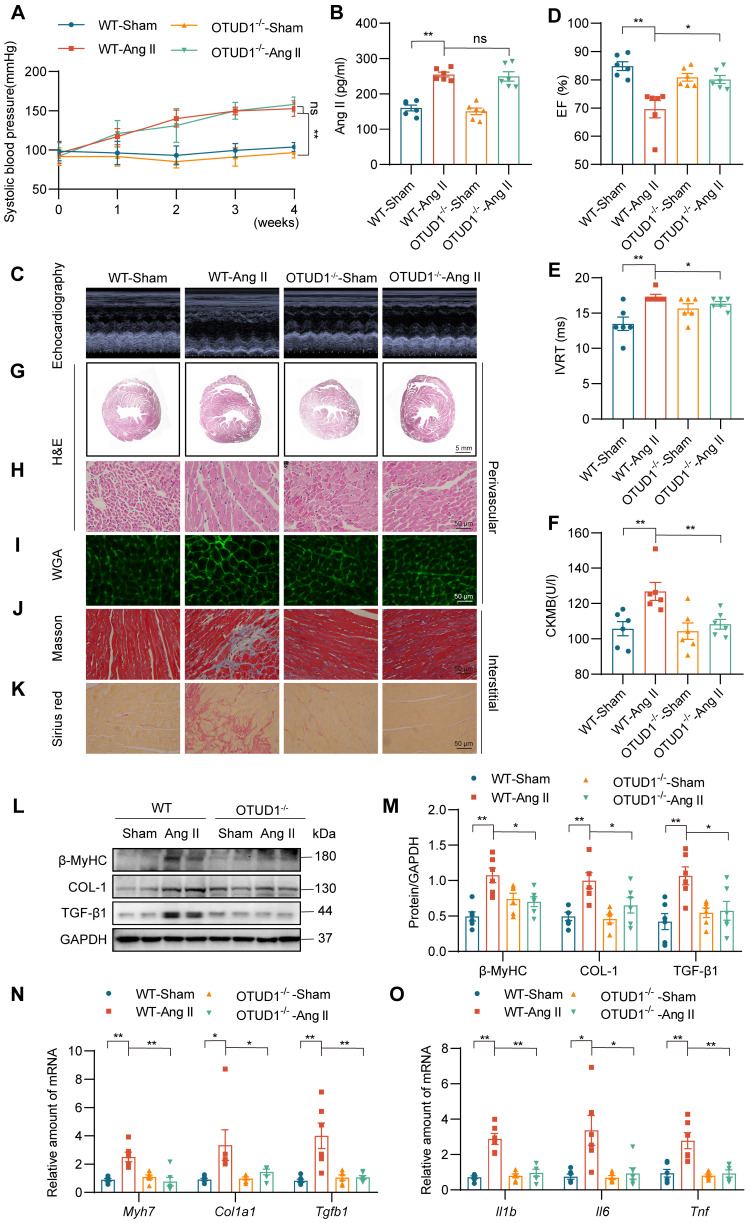
OTUD1 deficiency protects against Ang II-induced myocardial hypertrophy and fibrosis in mice. Wildtype and OTUD1 knockout mice were infused with saline (Sham) or Ang II for 4 weeks. **(A)** Systolic blood pressure was determined by non-invasive tail-cuff on a weekly basis. **(B)** Ang II levels were measured in serum samples from mice. **(C)** Representative echocardiographic images from mice in each group. **(D**, **E)** Cardiac functional tests showing ejection fraction (EF; panel D) and isovolumic relaxation time (IVRT; panel E). **(F)** Creatine kinase-MB (CK-MB) levels in serum of mice. **(G**, **H)** Hematoxylin and eosin (H&E) staining of heart tissues [scale bar = 5 mm (G) and 50 μm (H)]. **(I)** Staining of heart tissues with fluorophore-conjugated wheat germ agglutinin (WGA) was performed to measure cardiomyocyte hypertrophy [scale bar = 50 μm]. **(J**, **K)** Fibrosis in cardiac tissues was determined by Masson's Trichrome (J) and Picro Sirius Red (K) staining [scale bar = 50 μm]. **(L**, **M)** Western blot analysis of fibrosis-associated proteins β myosin heavy chain (β-MyHC), collagen 1(COL-1), and transforming growth factor- β1 (TGF-β1) in heart tissues. GAPDH was used as the loading control. Densitometric quantification is shown in panel M. **(N**, **O)** mRNA levels of fibrosis-associated genes (N) and inflammatory genes (O) in heart tissues of mice. Data was normalized to *Actb*. All quantitative data is presented as Mean ± SEM; n = 6; ns = not significant; * = *p* < 0.05 and ** = *p* < 0.01.

To examine the role of OTUD1 in cardiac dysfunction, we utilized OTUD1 knockout mice (Figure S1E-F). Wildtype and OTUD1 knockout mice were infused with saline or Ang II at 1 μg/kg/min for 4 weeks. Both wildtype and OTUD1 knockout mice infused with Ang II showed increased systolic blood pressure (Figure 2A) and increased levels of plasma Ang II (Figure 2B) when compared to saline-infused mice. These results suggest that OTUD1 deficiency does not affect blood pressure in response to Ang II. Echocardiography showed that OTUD1 deficiency prevents Ang II-induced cardiac dysfunction - evidenced by a lack of ejection fraction (EF) lowering and increases in isovolumetric relaxation time (IVRT) (Figure 2C-E, Table S3). Increases in heart weight: body weight and heart weight: tibia length was also not seen in OTUD1 knockout mice following Ang II infusion (Table S3). Serum levels of atrial natriuretic peptide (ANP) and creatine kinase-MB (CK-MB) were increased in wildtype mice infused with Ang II but not OTUD1 knockout mice (Figure 2F, Figure S2A).

Gross examination of heart tissues from mice suggested that OTUD1 knockout mice are protected against Ang II-induced cardiac hypertrophy (Figure S2B). H&E and WGA staining of the heart tissues also confirmed hypertrophic and structural changes in Ang II-challenged wildtype mice (Figure 2G-I, Figure S2C-F). These pathological and hypertrophic responses were not seen in OTUD1 knockout mice following Ang II challenge. Similarly, Picro Sirius Red and Masson's Trichrome staining revealed that OTUD1 knockout mice have reduced fibrosis compared to wildtype mice (Figure 2J-K, Figure S2G-L). Consistent with the histological examination and echocardiography, levels of fibrosis- and hypertrophy-associated factors cardiac myosin heavy chain beta (β-MyHC), collagen 1 (COL-1), and transforming growth factor-β1 (TGF-β1) were significantly lower in OTUD1 knockout mice following Ang II-infusion compared to wildtype mice (Figure 2L-N). In addition, mRNA levels of inflammatory genes were significantly increased in heart tissues of Ang II-infused wildtype mice (Figure 2O). Such induction was not seen in OTUD1 knockout mice. Together, these results show that OTUD1 deficiency protects against Ang II-induced myocardial hypertrophy and fibrosis.

### OTUD1 deficiency prevents TAC-induced myocardial hypertrophy and fibrosis

**Figure 3:**
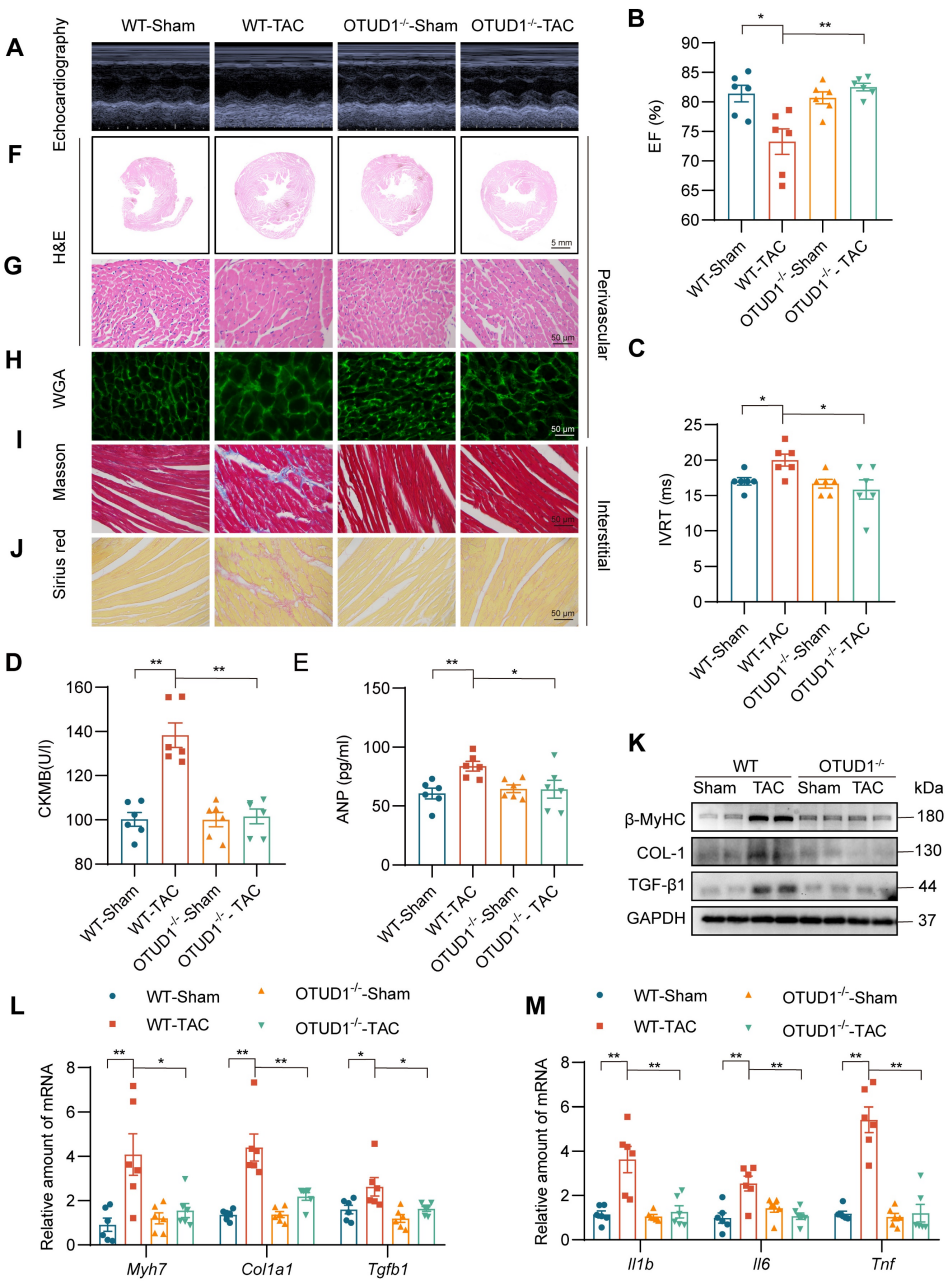
OTUD1 deficiency protects against TAC-induced myocardial hypertrophy and fibrosis in mice. Wildtype and OTUD1 knockout mice were subjected to transverse aortic constriction (TAC) to model pressure overload-induced cardiac hypertrophy and remodeling. Tissues from mice were analyzed at week 4 following TAC. **(A)** Representative echocardiographic images from mice in each group. **(B**, **C)** Cardiac functional tests showing ejection fraction (EF; panel B) and isovolumic relaxation time (IVRT; panel C). **(D**, **E)** CK-MB (D) and ANP (E) levels in serum of mice. **(F**, **G)** Hematoxylin and eosin (H&E) staining of heart tissues [scale bar = 5 mm (F) and 50 μm (G)]. **(H)** Staining of heart tissues with fluorophore-conjugated wheat germ agglutinin (WGA) was performed to measure cardiomyocyte hypertrophy [scale bar = 50 μm]. **(I**, **J)** Fibrosis in cardiac tissues was determined by Masson's Trichrome (I) and Picro Sirius Red (J) staining [scale bar = 50 μm]. **(K)** Western blot analysis of fibrosis-associated proteins in heart tissues. GAPDH was used as the loading control. **(L**, **M)** mRNA levels of fibrosis-associated genes (L) and inflammatory genes (M) in heart tissues of mice. Data was normalized to *Actb*. All quantitative data is presented as Mean ± SEM; n = 6; ns = not significant; * =* p* < 0.05 and ** = *p* < 0.01.

Building on our findings, we used the transverse aortic constriction (TAC) model of pressure overload-induced cardiac hypertrophy to study the role of OTUD1. Both wildtype and OTUD1 knockout mice were subjected to TAC and followed-up at 4 weeks. Echocardiography results showed that OTUD1 deficiency suppresses TAC-induced cardiac dysfunction (Figure 3A-C, Table S4). Similar to our Ang II mode, heart weight: body weight and heart weight: tibia length ratios were not elevated in OTUD1 knockout mice after TAC (Table S4). OTUD1 deficiency also prevented elevation in serum CK-MB and ANP levels in mice (Figure 3D-E). Histological analyses revealed that OTUD1 knockout mice are protected against TAC-induced cardiac hypertrophy and fibrosis (Figure 3F-J, Figure S3A-K). In agreement with these results, β-MyHC, COL-1 and TGF-β1 levels were increased in wildtype mice after TAC but such increases were not seen in OTUD1 knockout mice (Figure 3K-L, Figure S3L). As expected, we found that OTUD1 knockout mice do not show inflammatory gene induction after TAC (Figure 3M). these results confirm that OTUD1 deficiency suppresses deleterious myocardial hypertrophy and fibrosis.

### OTUD1 regulates cardiomyocyte hypertrophic and fibrotic responses in culture

**Figure 4:**
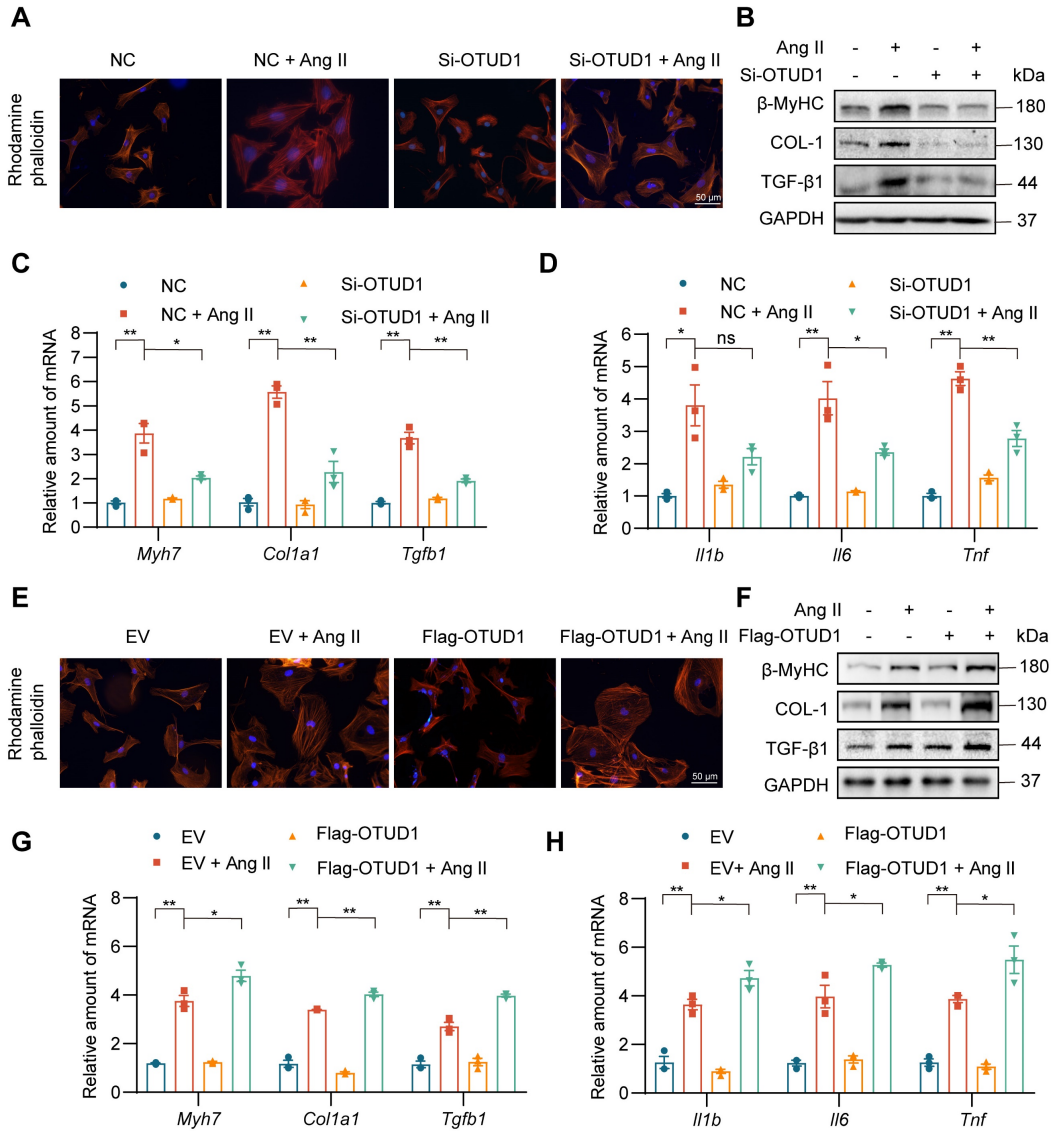
OTUD1 suppression reduces Ang II-induced cardiomyocyte hypertrophy and fibrogenic responses *in vitro*. Neonatal rat ventricular myocytes (NVRMs) were used to model Ang II effects in culture. Cells were transfected with siRNA against OTUD1 (panels A-D) or OTUD1 expressing vectors (panels E-H). Scrambled siRNA and empty vectors were used as control. Following transfections, cells were exposed to 1 μM Ang II for 48 h. **(A)** Following Ang II exposure, cells were stained with TRITC Phalloidin to assess hypertrophic responses [scale bar = 50 μm]. **(B)** Representative western blotting for β-MyHC, COL-1, and TGF-β1 in NVRMs. GAPDH was used as loading control. **(C**, **D)** mRNA levels fibrosis-associated genes (C) and inflammatory genes (D) were measured in NVRMs. Data was normalized to *Actb*. **(E)** Hypertrophic response to Ang II in cells expressing OTUD1. Figure showing TRITC Phalloidin staining [scale bar = 50 μm]. **(F)** Representative western blotting for β-MyHC, COL-1, and TGF-β1 in cells transfected with OTUD1 vector. **(G**, **H)** mRNA levels fibrosis-associated genes (G) and inflammatory genes (H) were measured in NVRMs. Data was normalized to *Actb*. All quantitative data is presented as Mean ± SEM; n = 3; ns = not significant; * = *p* < 0.05 and ** = *p* < 0.01.

To dissect the role of OTUD1 in cardiomyocyte responses, we first exposed primary cardiomyocytes (NVRMs) to 1 μM Ang II for different time periods and measured OTUD1 protein levels. Our data shows that Ang II induces the levels of OTUD1 within 60 min of exposure (Figure S4A-B). We showed the multiple siRNA sequences and transfections for siRNA experiments (Table S2, Figure S4C-D). In the subsequent experiments involving OTUD1 knockdown, we chose siRNA sequence #2. Consistent with previous study 22, Phalloidin staining showed that Ang II administration resulted in cell size increases compared with the control group, while OTUD1 knockdown prevents Ang II-induced cell size increases (Figure 4A, Figure S4E). This protective effect was also seen when we measured hypertrophy- and fibrosis-associated factors (Figure 4B-C, Figure S4F). As expected from our mouse modeling studies, Ang II failed to induce inflammatory genes in cardiomyocytes when OTUD1 was silenced (Figure 4D). We then expressed OTUD1 in NVRMs (Figure S4G-H) and performed similar experiments as with silencing. Our results show that OTUD1 expression aggravates hypertrophic and fibrotic responses of cardiomyocytes to Ang II (Figure 4E-H, Figure S4I-J). These results show that OTUD1 is needed for Ang II to induce hypertrophy and expression of fibrotic factors in cardiomyocytes.

### OTUD1 interacts directly with STAT3 and promotes STAT3 activation

**Figure 5:**
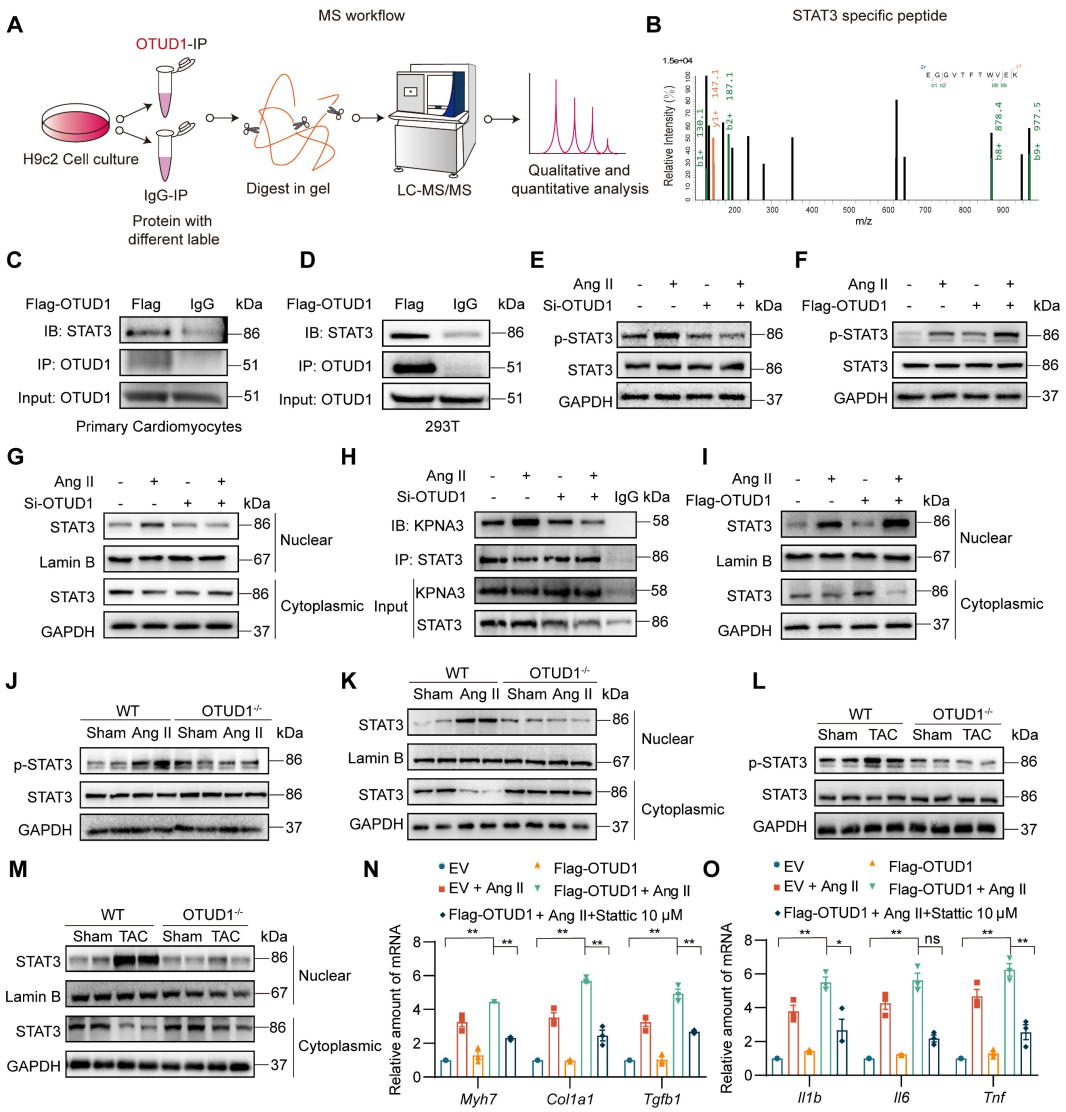
OTUD1 interacts directly with STAT3. **(A)** Schematic illustration of quantitative proteomic screen to identify proteins binding to OTUD1. **(B)** MS/MS spectrum of the peptide showing EGGVTFTWVEK from STAT3. Single-letter abbreviations: E, Glu; F, Phe; G, Gly; K, Lys; T, Thr; V, Val; W, Trp. m/z, mass/charge ratio. **(C**, **D)** NVRMs (C) and HEK-293T (D) were transfected with Flag-tagged OTUD1. Lysates from cells were immunoprecipitated with anti-OTUD1. STAT3 levels were detected by immunoblotting. IgG was used as control for IP. **(E**, **F)** NVRMs were transfected with siRNA against OTUD1 (E) or OTUD1-expressing vector (F). Cells were then exposed to 1 μM Ang II for 12 h. Levels of STAT3 phosphorylation (Tyr-705) were measured by immunoblotting. Total STAT3 and GAPDH were used for normalization. **(G)** OTUD1 siRNA transfected NVRMs were challenged with to 1 μM Ang II for 12 h. Levels of STAT3 in nuclear and cytosolic fractions were detected by immunoblotting. GAPDH and Lamin B were used as loading control. **(H)** NVRMs were treated as indicated in Panel G. STAT3 IP was performed and levels of KPNA3 (importin-α3) were detected by immunoblotting. IgG IP was used as control. **(I)** NVRMs were transfected with OTUD1-expressing vectors. Cells were then challenged with to 1 μM Ang II for 12 h. Levels of STAT3 in nuclear and cytosolic fractions were determined by immunoblotting. Lamin B and GAPDH were used as loading controls. **(J**, **K)** Levels of p-STAT3 (Tyr705) in whole heart lysates, and STAT3 in nuclear and cytosolic fractions of heart lysates of mice infused with Ang II for 4 weeks were detected by immunoblotting. **(L**, **M)** Levels of p-STAT3 (Tyr705) in whole heart lysates, and STAT3 in nuclear and cytosolic fractions of heart lysates of mice after TAC were detected by immunoblotting. **(N**, **O)** NVRMs were transfected with OTUD1-expressing vector (Flag-OTUD1) or empty vector (EV). Cells were then challenged with 1 μM Ang II for 12 h. Some cells were treated with STAT3 inhibitor Stattic. RNA levels of fibrosis-associated genes (N) and inflammatory genes (O) were measured. All quantitative data is presented as Mean ± SEM; n = 6 for Panels J-M and n = 3 for all other panels; ns = not significant; * = *p* < 0.05 and ** = *p* < 0.01.

Deubiquitinating enzymes modulate biological activities by affecting the degradation or function of substrate proteins. To identify potential substrate proteins regulated by OTUD1, we used OTUD1 immunoprecipitation coupled with mass spectrometry (Figure 5A). Of the potential OTUD1 binding proteins, STAT3 piqued our interest as it has been shown to play a pivotal role in modulating Ang II-induced hypertensive cardiac hypertrophy and remodeling upon abnormal activation 22. Representative MS spectra of STAT3 is shown in Figure 5B. To confirm this potential interaction between OTUD1 and STAT3, we transfected NVRMs and HEK-293T cells with Flag-tagged OTUD1 and showed that STAT3 interacts with OTUD1 following co-IP assay (Figure 5C-D).

Recent studies have shown increased tyrosine 705 phosphorylation of STAT3 by Ang II in mesangial cells and cardiomyocytes 23, 24. This increase is rapid and reaches maximum level at 12 h in primary cardiomyocytes, in the absence of serine 727 phosphorylation of STAT3 22. Based on these studies, we probed for Tyr705 p-STAT3 levels following OTUD1 modulation in cardiomyocytes. We first silenced OTUD1 in NVRMs and exposed the cells to 1 μM Ang II for 12 h. In this setting, OTUD1 knockdown prevented Ang II-induced p-STAT3 levels in cardiomyocytes (Figure 5E, Figure S5A). Conversely, expression of OTUD1 in cardiomyocytes exacerbated Ang II-induced p-STAT3 (Figure 5F, Figure S5B). Similar results were obtained in HEK-293T cells following OTUD1 expression (Figure S5C-D). In addition, as we can see, OTUD1 did not affect the expression level of STAT3 in cells (Figure 5E-F). These data show that OTUD1 functionally targets STAT3.

The process of STAT3 activation contains phosphorylation and nuclear translocation of STAT3. We then confirmed that STAT3 nuclear translocation is also inhibited following OTUD1 knockdown by measuring nuclear STAT3 levels in NVRMs (Figure 5G, Figure S5E). Nuclear translocation of STAT3 is facilitated by KPNA3 as noted in previous studies 25, 26. We tested the idea that OTUD1 binding to STAT3 may disrupt STAT3-KPNA3 association, which possibly reduces nuclear translocation. Our immunoprecipitation assay showed that Ang II increases STAT3-KPNA3 interaction, while this increased interaction was significantly inhibited after OTUD1 knockdown in cardiomyocytes (Figure 5H, Figure S5F). As expected, STAT3 nuclear translocation was increased upon OTUD1 over-expression in NVRMs (Figure 5I, Figure S5G). We then confirmed whether OTUD1 deficiency modulated STAT3 activation in hearts of Ang II-infused and TAC-induced mice. Analysis of heart tissue lysates showed that Ang II and TAC increased STAT3 phosphorylation and nuclear translocation in wildtype mice, but these measures of activity were suppressed in OTUD1 knockout mice (Figure 5J-M, Figure S5H-K).

Next, we explored the role of STAT3 in mediating the actions of OTUD1. We transfected NVRMs with Flag-OTUD1 to express OTUD1 and then challenged the cells with Ang II in the presence or absence of STAT3 inhibitor. We used Stattic to inhibit STAT3. Stattic selectively inhibits activation, dimerization, and nuclear translocation of STAT3 19, 27. In our experimental platform, Stattic reduced mRNA levels of inflammatory genes and hypertrophy- and fibrosis-associated genes in cardiomyocytes upon Ang II exposure, even in the presence of OTUD1 expression (Figure 5N-O). These *in vitro* studies show that OTUD1 interacts with STAT3 and regulates Tyr-705 phosphorylation and nuclear translocation to module expression of cardiac remodeling genes.

### OTUD1 regulates the activity of STAT3 through deubiquitination

**Figure 6:**
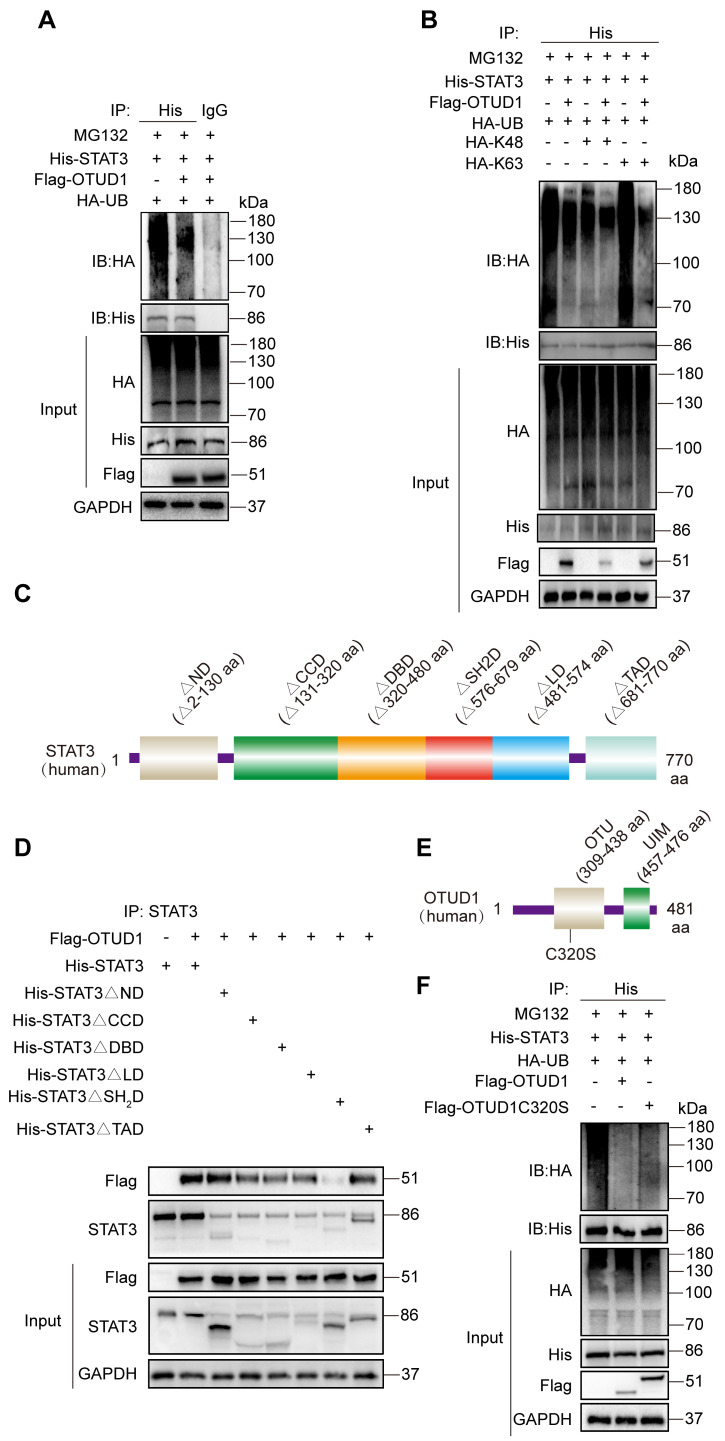
OTUD1 regulates the activity of STAT3 protein through deubiquitination. **(A)** HEK-293T cells were transfected with His-STAT3, HA-UB and Flag-OTUD1. Cells were then exposed to MG132. Ubiquitinated STAT3 was detected by immunoblotting using an His-specific antibody [control = IgG]. **(B)** HEK-293T cells were transfected with His-STAT3, HA-UB, HA-K48, HA-K63 and Flag-OTUD1. Cells were then exposed to MG132. Ubiquitinated STAT3 was detected by immunoblotting. **(C)** Schematic illustration of the STAT3 deletion constructs. **(D)** HEK-293T cells were transfected with His-tagged full length STAT3, various STAT3 constructs, and Flag-tagged OTUD1. Anti-His antibody was used for IP. Levels of Flag-tagged OTUD1 were detected by IB. **(E)** Schematic illustration of OTUD1 active site deletion construct. **(F)** Immunoprecipitation of His-STAT3 in HEK-293T cells that co-expressed HA-UB, Flag-OTUD1 and Flag-OTUD1C320. Ubiquitinated STAT3 was detected by immunoblotting.

To characterize the mechanism by which OTUD1 regulates STAT3 activity, we co-transfected STAT3 (His-tagged) and OTUD1 (Flag-tagged) plasmids in HEK-293T cells. We then treated cells with MG132 to prevent proteasomal degradation of STAT3 protein. From this setup, we show that OTUD1 reduces the ubiquitination of STAT3 (Figure 6A). We then co-transfected STAT3, OTUD1, and mutated ubiquitin plasmids retaining only K48 and K63 active sites in HEK-293T cells and exposed the cells to MG132. We observed that the HA-K63 plasmid was sufficient to reduce ubiquitination of STAT3 in the presence of OTUD1, at levels comparable to HA-Ub (Figure 6B).

To identify STAT3 sites for OTUD1 interaction, we transfected cells with various STAT3 constructs. STAT3 has six domains including the N Domain (ND), Coil-coil Domain (CCD), DNA Binding Domain (DBD), Linker Domain (LD), SH2 Domain (SH2D), and TAD Domain (TAD). We generated six STAT3 domain truncation mutants (Figure 6C). Through co-transfections of OTUD1 and mutated STAT3 plasmids in HEK-293T cells, we determined that STAT3 could not bind OTUD1 when amino acids 576 to 679 were missing (Figure 6D). These results indicate that STAT3 binds to OTUD1 directly via STAT3 SH2 domain. After identifying STAT3-OTUD1 interaction site, we mutated active sites on OTUD1 (cysteine at position 320 to serine) (Figure 6E). We found that OTUD1C320S could no longer remove ubiquitin molecules from STAT3 (Figure 6F). These results demonstrate that the cysteine at position 320 of OTUD1 is implicated in the removal of ubiquitin molecules from STAT3, thereby preventing its degradation.

### OTUD1 increases Ang II-induced cardiomyocyte myocardial hypertrophy and fibrosis by regulating STAT3

**Figure 7:**
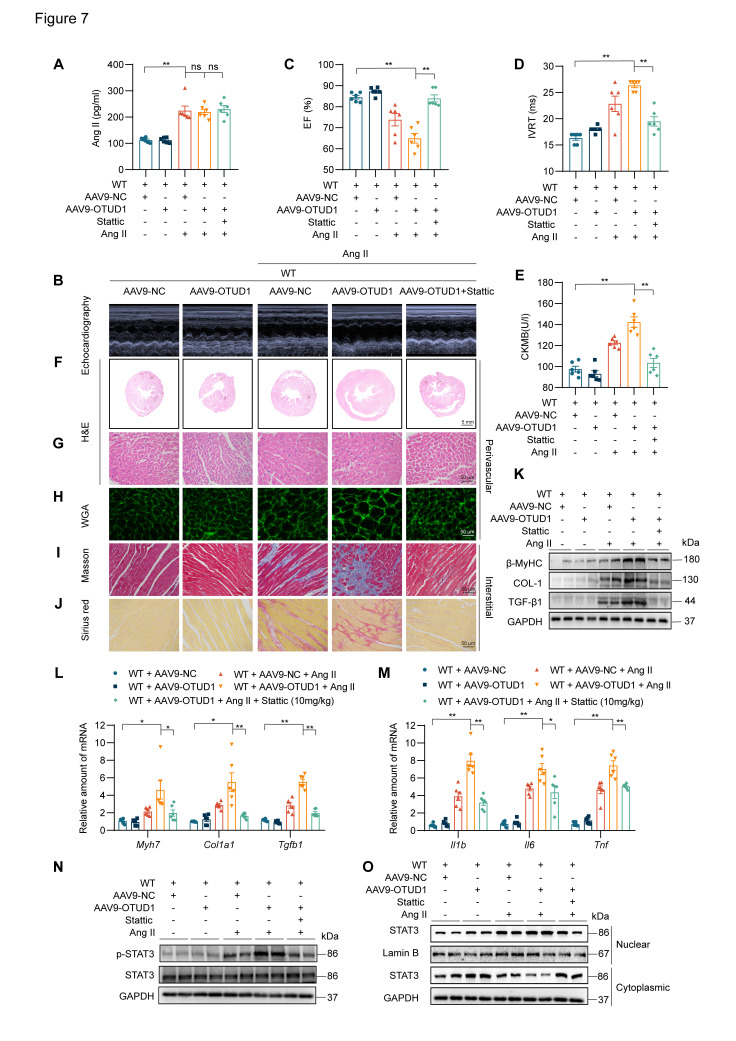
OTUD1 expression protects against Ang II-induced cardiac hypertrophy and fibrosis by regulating STAT3. C57BL/6 mice received two injections of AAV9 encoding OTUD1, one month apart. Saline or Ang II was then infused for 2 weeks. **(A)** Ang II levels in serum samples obtained from mice. **(B)** Representative echocardiographic images from mice in each experimental group. **(C**, **D)** Cardiac functional tests showing ejection fraction (EF; panel C) and isovolumic relaxation time (IVRT; panel D). **(E)** CK-MB levels in serum obtained from mice. **(F, G)** Hematoxylin and eosin (H&E) staining of heart tissues [scale bar = 5 mm (F) and 50 μm (G)]. **(H)** Staining of heart tissues with fluorophore-conjugated wheat germ agglutinin (WGA) to measure cardiomyocyte hypertrophy [scale bar = 50 μm]. **(I**, **J)** Fibrosis in cardiac tissues was determined by Masson's Trichrome (I) and Picro Sirius Red (J) staining [scale bar = 50 μm]. **(K)** Western blot analysis of fibrosis-associated proteins β-MyHC, COL-1, and TGF-β1 in heart tissues. GAPDH was used as the loading control. **(L**, **M)** mRNA levels of fibrosis-associated genes (L) and inflammatory genes (M) in heart tissues of mice. Data was normalized to *Actb*. **(N**, **O)** Levels of p-STAT3 (Tyr705) in whole heart lysates, and nuclear and cytosolic STAT3 in fractionated heart lysates from mice. All quantitative data is presented as Mean ± SEM; n = 6; ns = not significant; * = *p* < 0.05 and ** = *p* < 0.01.

Our last objective was to examine if STAT3 is a functional substrate of OTUD1 in hearts and mediates OTUD1-promoted cardiac remodeling in mice. To achieve this goal, we constructed AAV9-encoding OTUD1 particles and administered them to mice via tail vein injection. Mice were then infused with saline or Ang II for 2 weeks. We also treated some mice with STAT3 inhibitor Stattic. A brief flow chart of this experiment is shown in Figure S6. AAV9 infection was successful and showed increased OTUD1 expression in heart tissues of mice (Figure S7A-B). As expected, Ang II levels were increased in all mice in which Ang II was infused (Figure 7A) and caused increased systolic blood pressure (Figure S7C). Cardiac functional tests revealed that OTUD1 expression in the heart increases Ang II-induced cardiac dysfunction (Figure 7B-7D, Table S5). This exaggerated response to Ang II was prevented in mice treated with STAT3 inhibitor Stattic. Supporting these results are levels of ANP and CK-MB which were increased in OTUD1-expressing heart tissues and reduced following Stattic treatment (Figure 7E, Figure S7D).

Comprehensive histological analyses showed that OTUD1 expression enhances Ang II-responses manifesting as cardiac hypertrophy and fibrosis (Figure 7F-J, Figure S7E-O). In these analyses, we also found that Stattic is able to prevent these pathological changes in heart tissues. Consistent with these results, hypertrophic and fibrotic factors were increased in mice with cardiac OTUD1 expression and Ang II challenge (Figure 7K-O, Figure S8A). As expected, we found that inflammatory gene were increased in mice with cardiac OTUD1 expression and Ang II challenge, while STAT3 inhibition was able to prevent these inductions (Figure 7M). Lastly, we probed for p-STAT3 levels and nuclear STAT3 translocation in lysates prepared from heart tissues of mice. Results showed that OTUD1 expression increases Ang II-mediated p-STAT3 and STAT3 nuclear translocation and that Stattic treatment prevents these measures of STAT3 activation (Figure 7N-O, Figure S8B-C). These results demonstrate that OTUD1 promotes Ang II-induced cardiac remodeling and dysfunction through activating STAT3.

## Discussion

This study set out to investigate DUBs in hypertensive heart failure, and revealed increased OTUD1, an OUT domain-containing DUB member, in the myocardium in response to Ang II administration. OTUD1 knockout mice, when challenged with the same Ang II regimen, showed suppressed myocardial hypertrophy and cardiac dysfunction. We replicated these results in the TAC model of cardiac pressure overload. Mechanistically, we showed that OTUD1 binds to STAT3 and causes deubiquitination of STAT3. We also demonstrated that cysteine at position 320 of OTUD1 exerts K63 deubiquitination to promote STAT3 phosphorylation and nuclear translocation. Our key findings are summarized in the Graphical Abstract.

Most DUBs are cysteine proteases 28. Almost a 100 putative DUBs have been identified in humans. Research on DUBs in maladaptive cardiac remolding is scarce. Only recently, a few DUBs have been identified in various models of cardiac dysfunction. For example, Wang and colleagues identified upregulated cyclindromatosis (CYLD) in TAC-induced cardiac dysfunction 29. They also showed that CYLD knockout mice fail to show TAC-induced cardiac hypertrophy and results obtained from echocardiography. Conversely, a few DUBs have been shown to be protective in heart disease. Using a similar aortic constriction model, increased expression of A20/tumor necrosis factor alpha induced protein 3 (TNFAIP3) was noted 30, 31. A20 was also induced in neonatal cardiomyocytes when the cells were exposed to phenylephrine and endothelin 1 30. However, in these A20 studies, overexpression of A20 improved cardiac function and inhibited cardiac remodeling and cell apoptosis. He and colleagues identified decreased levels of USP4 in human failing hearts and in murine hypertrophied hearts 32. They also demonstrated that deficiency of endogenous USP4 promotes myocyte hypertrophy induced by Ang II *in vitro*. This suggests that Ang II, in addition to increasing OTUD1, may supress USP4. Th collective action of both DUBs may be important to the induction is deleterious cardiac hypertrophy, fibrosis, and the induction of inflammatory factors. This is certainly a future avenue worth exploring.

Although DUBs have been reported to have a range of redundant substrates, studies also suggest specificity. For example, OTUD1 has been shown to enhances iron transport and potentiate antitumor immunity in colon cancer by targeting/regulating iron-responsive element-binding protein 2 33. In our molecular studies, we identified STAT3 as one of the substrates of OTUD1 in the heart. STAT3 has been reported to induce a broad range of cellular and molecular activities that play roles in cardiac (patho) physiology 34, 35. Cardiomyocyte-specific expression of STAT3 leads to spontaneous concentric cardiac hypertrophy 36. Other studies have also shown that activating STAT3 via LIF induced hypertrophic growth in cultured cardiomyocytes and in cardiomyocytes in mice 37. In addition to hypertrophy, STAT3 may also mediate inflammation in the heart 38, 39. We have recently reported that STAT3 is activated in both Ang II- and TAC-induced cardiac hypertrophy and fibrosis 22. Although the mechanisms of STAT3 activation in cardiac dysfunction are not fully clear, one important mechanism may be decreased ubiquitination and increased protein stability by OTUD1, as noted in our present study.

STAT3 belongs to the transcription factor family. Upon stimulation, cytoplasmic STAT3 is phosphorylated, dimerizes, and then translocates to the nucleus to regulate the transcription of target gene 40. Two phosphorylating sites, Y705 and Y727, constitute an “ignition” for canonical STAT3 activation 41. Y705 phosphorylation of STAT3 leads to STAT3 dimer formation followed by translocation to the nucleus and induction of gene expression. Our previous study has also showed that Ang II induces STAT3 phosphorylation at Y705, rather than Y727, in cardiomyocytes to promote pathological cardiac remodeling 22. STAT3 protein has been reported to be modified through post-transcriptional ubiquitination, which further promotes STAT3 stabilization and activation. For example, Jonathan J. Cho et al found that Hectd3 mediates K27-linked polyubiquitin chains on STAT3 and identified K180, in the coiled-coil domain (CCD), as the target for polyubiquitination, which was important for proper STAT3 Y705 phosphorylation in Th17 cells 42. Another study found that tumor necrosis factor receptor-associated factor 6 (TRAF6) mediated K63-linked ubiquitination of STAT3 at six lysine residues in the SH2 domain to promote STAT3 Y705 phosphorylation in mouse embryonic fibroblasts 43, 44. In addition, STAT3 has been identified as a substrate of the E3 ubiquitin ligase COP1, which also contributes to STAT3 phosphorylation at Tyr705, in prostate epithelial cells 45. Regarding to the deubiquitination modification of STAT3, however, only a DUB, USP28, was found to interacts with STAT3 and deubiquitinates STAT3 in a K48-linked manner, which leads to STAT3 stabilization. So far, there is no DUBs reported to deubiquitinate STAT3 in K63-linked way and regulate Y705 phosphorylation. In this study, we found that OTUD1 deubiquitinates STAT3 in a K63-linked way to promote Y705 phosphorylation and nuclear translocation. Although the involved lysine residues in STAT3 are not identified, this is the first time to present a K63-linked deubiquitination of STAT3 by OTUD1 to regulate STAT3 activity. This study provides a new post-transcriptional modification of STAT3 in the research field of STAT3 biology.

Although our study focused on STAT3 as an OTUD1 substrate, it will be important to determine what other substrates, regulated by OTUD1, may be involved in cardiac remodeling. Studies conducted in cancer models provide exciting possibilities. In breast cancer cells, OTUD1 deubiquitinates TGF-β1 inhibitor SMAD7 and increases TGFB receptor 1 turnover 46. In this model, OTUD1 represses breast cancer metastasis by mitigating TGF-β activity and loss of OTUD1 in aggressive forms derives TGF-β signaling and metastasis. However, in our study, we noted increased TGF-β1 ligand expression in cultured cardiomyocytes exposed to Ang II, and in heart tissues of mice challenged with Ang II and TAC. We further show that TGF-β1 is not induced when OTUD1 is deficient *in vitro* and *in vivo*. Therefore, assessment of TGF-β1 signaling activity would be helpful in determining whether OTUD1-mediated regulation of TGF-β1 is specific to breast cancer or is also evident in cardiac remodeling but at the activity level. Another cancer study worth noting is by Luo et al 47. These researchers identified apoptosis-inducing factor (AIF) as a substrate of OTUD1 in esophageal cancer. Specifically, they showed that OTUD1-mediated deubiquitination of AIF at K244 disrupts the normal mitochondrial structure and compromises oxidative phosphorylation, and deubiquitination at K255 promotes parthanatos. They also showed that OTUD1 stabilizes DDB1 and CUL4 associated factor 10 to induce caspase-dependent apoptosis of cancer cells. Although we did not specifically measure cell death in our *in vitro* and *in vivo* models, it could be an interesting future study because we know that cardiomyocyte damage and loss occurs in patients with hypertensive heart disease 48.

In conclusion, the current study identified upregulated OTUD1 in Ang II-challenged model of hypertensive cardiac dysfunction and showed that Ang II- and TAC-induced cardiac hypertrophy, fibrosis, and functional deficits are essentially prevented when OTUD1 is knocked out. Conversely, increased expression of OTUD1 enhances Ang II-induced injuries in the heart. In mechanistic studies, we identified STAT3 as an important OTUD1 substrate. We mapped SH2 domain on STAT3 as the OTUD1 binding site and showed that OTUD1 deubiquitinates STAT3 to increase p-STAT3 levels and STAT3 nuclear translocation. These studies have discovered OTUD1 as an important factor in hypertensive cardiac disease through directly regulating STAT3 activity.

## Supplementary Material

Supplementary figures and tables.

## Abbreviations

Ang II: Angiotensin II
ANP: Atrial natriuretic peptide
CCD: Coil-coil Domain
CKMB: creatine kinase-MB
COL1A1: collagen type I alpha 1 chain
DBD: DNA Binding Domain
DUBs: deubiquitinases
EF: ejection fraction
H&E: hematoxylin and eosin
IL-1: Interleukin 1
IL-6: Interleukin-6
IVRT: isovolumetric relaxation time
KPNA3: importin-α3
LD: Linker Domain
MYH7: myosin heavy chain 7
ND: N Domain
NRVMs: neonatal rat ventricular myocytes
OTUD1: OTU Domain-Containing Protein 1
RAS: renin-angiotensin system
SH2D: SH2 Domain
STAT3: signal transducer and activator of transcription 3
TAC: transverse aortic constriction
TAD: TAD Domain
TGF-β1: transforming growth factor beta 1
TNF: Tumor necrosis factor
Ub: ubiquitin
WGA: wheat germ agglutinin
