# Supplementary material for: OTUD1 promotes pathological cardiac remodeling and heart failure by targeting STAT3 in cardiomyocytes
Source: Theranostics. 2023 Apr 17;13(7):2263–80. doi: 10.7150/thno.83340 (PMC10157730; doi:10.7150/thno.83340)
Supplement: Supplementary file 1 — Supplementary figures and tables. [file thnov13p2263s1.pdf]

OTUD1 promotes pathological cardiac remodeling and heart failure by targeting STAT3 in cardiomyocytes

Short title: OTUD1 promotes cardiac remodeling

Supplemental (Online) File: 5 Tables, and 8 Figures

Table S1: Primer sequences for real-time qPCR assay and genotyping.

Gene	Species	Sequence
Actb	Rat	AAGTCCCTCACCCTCCCAAAAG<br>AAGCAATGCTGTCACCTTCCC
Col1a1	Rat	GACATCCCTGAAGTCAGCTGC<br>TCCCTTGGGTCCCTCGAC
Il1	Rat	CACCTCTCAAGCAGAGCACAG<br>GGGTTCCATGGTGAAGTCAAC
Il6	Rat	GAGTTGTGCAATGGCAATTC<br>ACTCCAGAAGACCAGAGCAG
Myh7	Rat	GAGGAGAGGGCGGACATT<br>ACTCTTCATTCAGGCCCTTG
Tgfb1	Rat	GCAACAACGCAATCTATGAC<br>CCTGTATTCCGTCTCCTT
Tnf	Rat	TACTCCCAGGTTCTCTTCAAGG<br>GGAGGCTGACTTTCTCCTGGTA
Actb	Mouse	CCGTGAAAAGATGACCCAGA<br>TACGACCAGAGGCATACAG
Col1a1	Mouse	TGGCCTTGGAGGAAACTTTG<br>CTTGGAAACCTTGTGGACCAG
Il1	Mouse	TCGCAGCAGCACATCAACAAGAG<br>AGGTCCACGGGAAAGACACAGG
Il6	Mouse	GAGGATACCACTCCCAACAGACC<br>AAGTGCATCATCGTTGTTCATACA
Myh7	Mouse	CAAAGGCAAGGCAAAGAAAG<br>TCACCCCTGGAGACTTTGTC
Tgfb1	Mouse	TGACGTCACTGGAGTTGTACGG<br>GGTTCATGTCATGGATGGTGC
Tnf	Mouse	TGATCCGCGACGTGGAA<br>ACCGCCTGGAGTTCTGGAA
Otud1 (KO genotyping)	Mouse	ATGCAGCTCTACAGCAGCGT<br>GTGAGCCTCGGCTTCGGGAT
Otud1	Human	CCCACGGTGTCTACCATGATTCA<br>CTTCGTCGCGTTTCCTTTGCA
Actb	Human	CCTGGCACCCAGCACAAT<br>GCCGATCCACACGGAGTACT

Table S2: siRNA sequences for OTUD1 knockdown in neonatal rat ventricular myocytes

siRNA	Species	Sequence
OTUD1 #1	Rat	5'-CUGACGGUAACUGCCUUUATT-3'<br>5'-UAAAGGCAGUUACCGUCAGTT-3'
OTUD1 #2	Rat	5'-GGUGCAAGCAAACCCAAAUTT-3'<br>5'-AUUUGGGUUUGCUUGCACCTT-3'
OTUD1 #3	Rat	5'-GCCUAGUAUUUGGCUUAGUTT-3'<br>5'-ACUAAGCCAAAUACUAGGCTT-3'
Negative control (NC)	Rat	5'-UUCUCCGAACGUGUCACGUTT-3'<br>5'-ACGUGACACGUUCGGAGAATT-3'

Table S3: Biometric and echocardiographic parameters in Ang II-challenged mice.

Model 1 (Ang II model)	Sham		Continuous Ang II Pump Infusion	
	WT	OTUD$^{-/-}$	WT	OTUD$^{-/-}$
	n=6	n=6	n=6	n=6
EF, %	84.84±0.04	80.92±0.03	69.66±0.08**	80.10±0.03#
FS, %	47.85±0.05	43.50±0.03	33.87±0.05**	43.27±0.03##
HR, bpm	615.00±48.27	609.33±20.92	592.33±43.98	590.17±52.30
LVIDd, mm	2.63±0.40	2.73±0.51	2.93±0.30	2.51±0.30#
IVSD, mm	0.57±0.05	0.68±0.04	0.73±0.05**	0.67±0.05#
PWD, mm	0.58±0.04	0.68±0.04	0.7±0.06**	0.6±0.08
E wave, m/s	0.95±0.11	0.71±0.17	0.73±0.38	0.68±0.13
Tei Index	0.71±0.01	0.70±0.11	0.92±0.16*	0.76±0.12
IRT, ms	13.5±2.3	15.67±1.63	17.33±0.8**	16.33±0.82#
HW/BW, mg/g	5.63±0.09	5.86±0.55	6.51±0.66**	5,83±0.34#
HW/TL, mg/mm	7.30±0.42	7.33±0.54	7.75±0.19*	7.12±0.30##

Transthoracic echocardiography was performed on mice at the end of the animal study. Ang II = angiotensin II; BW = body weight; EF = ejection fraction; E wave = peak mitral E velocity; FS = fractional shortening; HR = heart rate; HW = heart weight; IVRT = isovolumic relaxation time; IVSD = diastole interventricular septal thickness; LVIDd = diastole left ventricle internal dimension; PWD = diastole posterior wall thickness; Tei index = a myocardial performance index. Data presented as Mean ± SEM; * = $p < 0.05$ and ** = $p < 0.01$ compared to WT-Sham; # = $p < .05$ and ## = $p < 0.01$ compared to WT + Ang II.

Table S4: Biometric and echocardiographic parameters in TAC-challenged mice.

Model 2 (TAC model)	Sham		TAC	
	WT	OTUD1$^{-/-}$	WT	OTUD1$^{-/-}$
	n=6	n=6	n=6	n=6
EF, %	81.41±0.03	80.69±0.02	73.27±0.05*	82.54±0.02##
FS, %	43.90±0.04	42.32±0.04	36.92±0.05*	44.82±0.02##
HR, bpm	538.50±32.67	523.67±27.87	532.67±37.13	530.50±28.46
LVIDd, mm	2.5±0.54	2.56±0.18	2.93±0.27	2.53±0.26#
IVSD, mm	0.63±0.05	0.70±0.09	0.90±0.09**	0.78±0.04#
PWD, mm	0.63±0.05	0.70±0.09	0.90±0.09**	0.72±0.04##
E wave, m/s	0.62±0.09	0.66±0.07	0.66±0.17	0.62±0.06
Tei Index	1.48±0.15	1.43±0.09	1.87±0.18**	1.44±0.11##
IRT, ms	17.00±1.26	16.67±1.51	20±2.10*	15.83±3.37#
HW/BW, mg/g	4.66±0.29	4.92±0.19	6.11±0.73**	4.90±0.30##
HW/TL, mg/mm	5.13±0.21	5.42±0.23	6.88±0.78**	5.89±0.32#

Transthoracic echocardiography was performed on mice at the end of the animal study. BW = body weight; EF = ejection fraction; E wave = peak mitral E velocity; FS = fractional shortening; HR = heart rate; HW = heart weight; IVRT = isovolumic relaxation time; IVSD = diastole interventricular septal thickness; LVIDd = diastole left ventricle internal dimension; PWD = diastole posterior wall thickness; Tei index = a myocardial performance index. Data presented as Mean ± SEM; * = $p < 0.05$ and ** = $p < 0.01$ compared to WT-Sham; # = $p < .05$ and ## = $p < 0.01$ compared to WT + TAC.

Table S5: Biometric and echocardiographic parameters in Ang II-challenged mice treated with OTUD1-AAV9.

Model 3 (Ang II model)	WT+AAV-NC	WT+AAV-OTUD1	Continuous Ang II Pump Infusion		
			WT + AAV-NC	WT + AAV9-OTUD1	WT+AAV9 -OTUD1 +Static (10mg/kg)
	n=6	n=6	n=6	n=6	n=6
EF, %	84.5±0.02	87.2±0.02	73.7±0.07	64.9±0.06*	83.9±0.04##
FS, %	47.3±0.02	50.3±0.02	36.9±0.06	30.0±0.04*	47.7±0.05##
HR, bpm	534±25.91	534.2±27	548.8±36.22	527±37.45	547.5±38.5
LVIDd, mm	1.45±0.10	1.28±0.14	2.65±0.10**	2.76±0.10	2.00±0.35##
IVSD, mm	0.68±0.04	0.82±0.17	1.1±0.09	1.15±0.08ns	0.75±0.05##
PWD, mm	0.68±0.04	0.72±0.08	0.97±0.10	1.11±0.10*	0.72±0.04##
E wave, m/s	0.72±0.16	0.76±0.07	0.61±0.02	0.55±0.06*	0.73±0.06##
Tei Index	0.70±0.02	1.01±0.27	1.21±0.10	1.53±0.34*	1.14±0.19#
IRT, ms	16.33±1.03	18±0.63	22.83±3.60	26.33±1.03*	19.5±2.17##
HW/BW, mg/g	4.29±0.27	4.32±0.31	5.02±0.11	5.46±0.22**	4.59±0.29##
HW/TL, mg/mm	5.62±0.42	5.56±0.38	6.46±0.13	6.80±0.05**	5.78±0.41##

Transthoracic echocardiography was performed on mice at the end of the animal study. Ang II = angiotensin II; BW = body weight; EF = ejection fraction; E wave = peak mitral E velocity; FS = fractional shortening; HR = heart rate; HW = heart weight; IVRT = isovolumic relaxation time; IVSD = diastole interventricular septal thickness; LVIDd = diastole left ventricle internal dimension; PWD = diastole posterior wall thickness; Tei index = a myocardial performance index. Data presented as Mean ± SEM; * = $p < 0.05$ and ** = $p < 0.01$ compared to WT-Sham; # = $p < .05$ and ## = $p < 0.01$ compared to WT + Ang II.

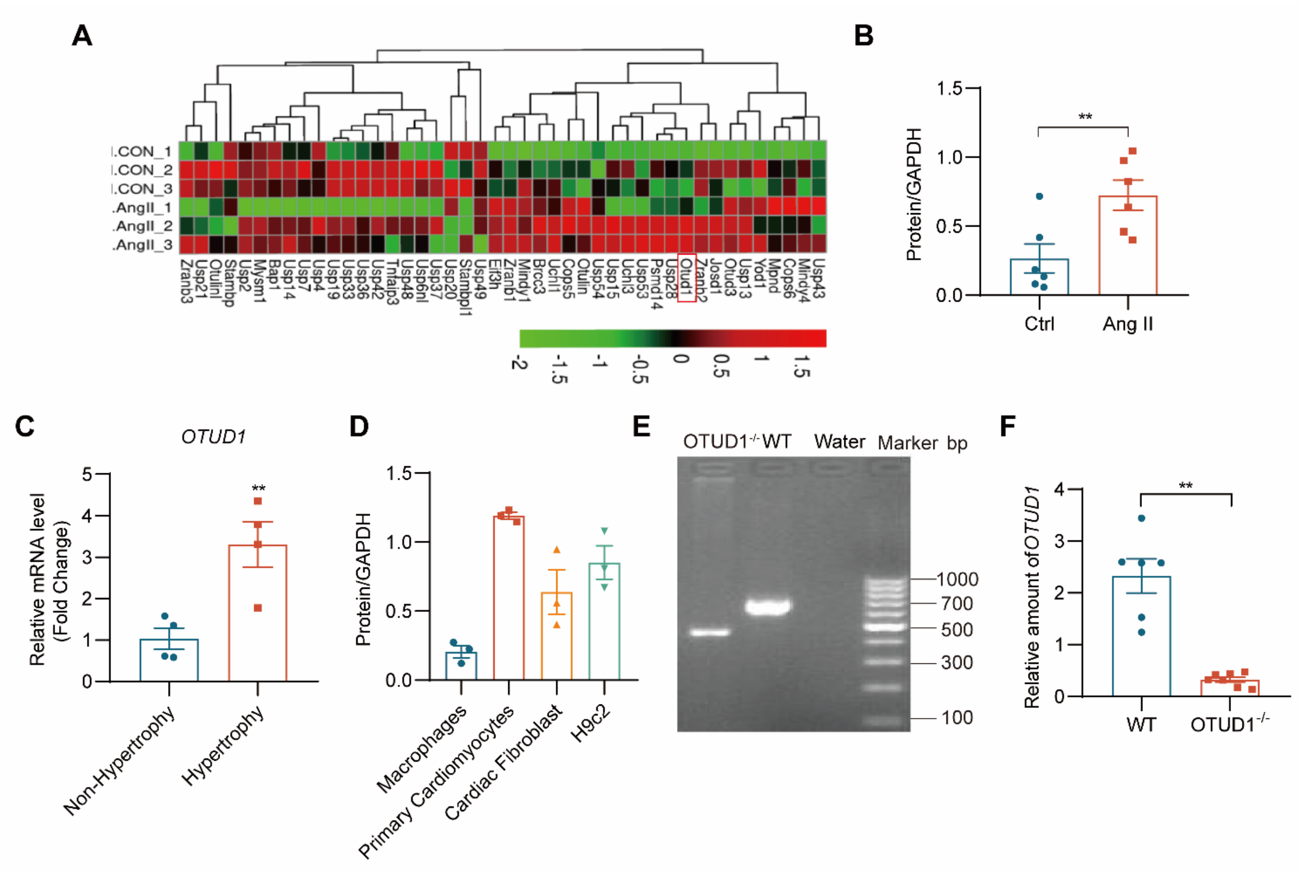

Figure S1: Heatmap showing deubiquitinating enzymes in heart tissues of Ang II-challenged mice.

(A) We performed RNA-sequencing analysis using the wildtype B6 mice (Ctrl) and mice challenged with Ang II for 4 weeks. Figure showing heatmap of the top 44 differentially expressed deubiquitinating enzymes. Cut-off was set as log2 > 1.5 ($p<0.05$). **(B)** Densitometric quantification of blots in Figure 1B. **(C)** Real-time qPCR analysis of the mRNA expression of OTUD1 in hypertrophic myocardial tissues of heart failure patients and normal human heart tissues. n = 4 per group. The mRNA level from each group was normalized to one value from the control/non-hypertrophy group, which was set to 1. Data were normalized to *Actb*. All quantitative data are presented as Mean ± SEM; ** = $p < 0.01$. **(D)** Densitometric quantification of blots in Figure 1D. **(E)** Genotyping of wildtype (WT) and OTUD1 knockout mice ($OTUD1^{-/-}$). Genotyping primers detected 690 bp product in WT mice and <500 bp product in OTUD1 knockout mice. **(F)** mRNA level of *OTUD1* in hearts of wild-type mice and $OTUD1^{-/-}$ mice determined by RT-qPCR. Transcripts were normalized to *Actb* (n=6 per group).

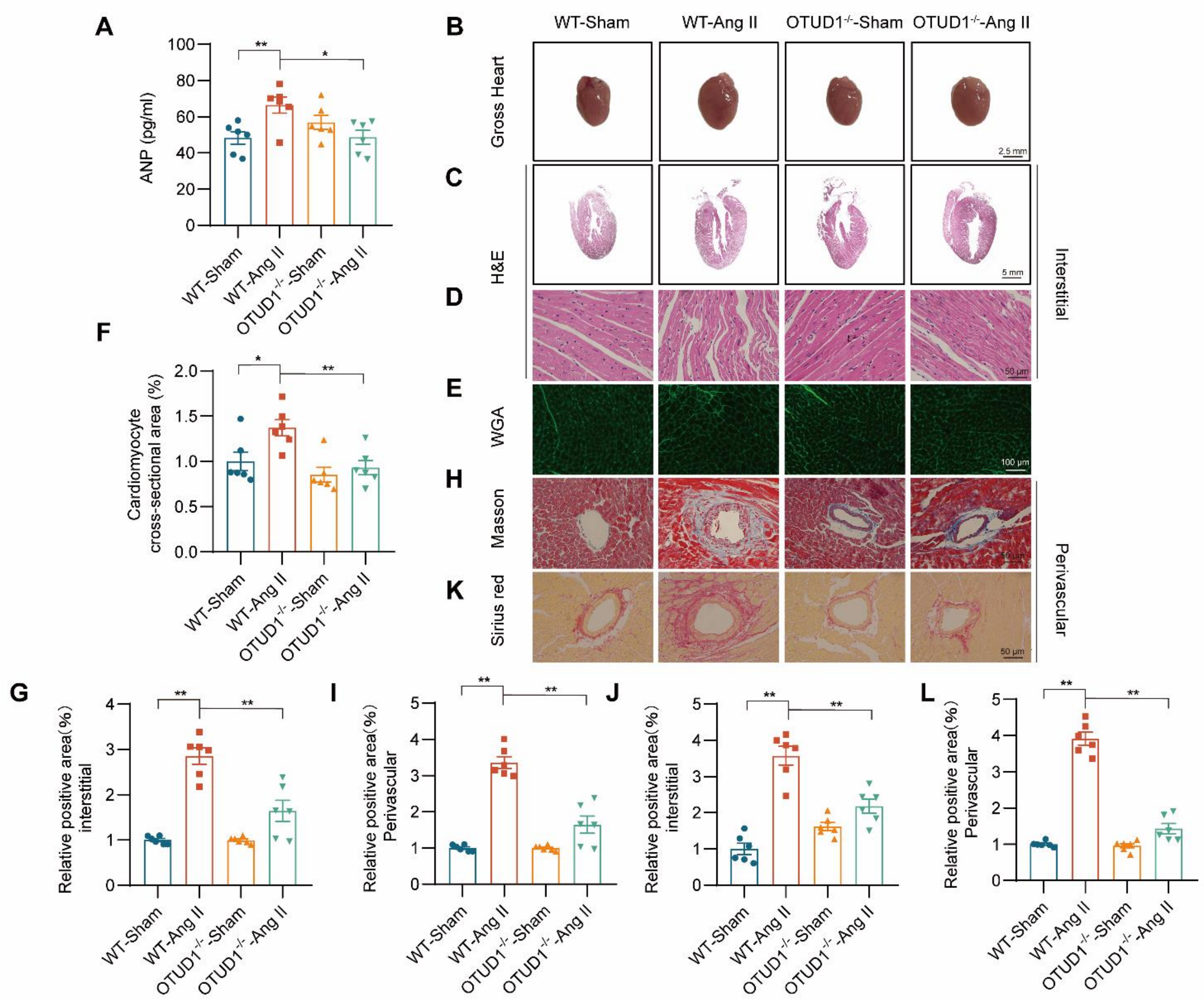

Figure S2: OTUD1 deficiency prevents Ang II-induced myocardial hypertrophy and fibrosis. (A) Atrial natriuretic peptide (ANP) levels in serum of wildtype (WT) and OTUD1 knockout mice, infused with saline (Sham) or Ang II for 4 weeks. **(B)** Representative whole heart images [scale bar = 2.5 mm]. **(C**, **D)** Hematoxylin and eosin-stained heart tissues [scale bar = 5 mm (C) and 50 μm (D)]. **(E)** Heart tissues were stained with wheat germ agglutinin (WGA) to assess cell size changes [scale bar = 100 μm]. **(F)** Quantification of cardiomyocyte size following WGA staining. **(G)** Interstitial fibrosis was quantified from Masson's Trichrome staining of heart tissues. Representative stained images are shown in Figure 2J. **(H**, **I)** Perivascular fibrosis was detected by staining heart tissues with Masson's trichrome (H) [scale bar = 50 μm]. Quantification is shown in panel I. **(J)** Quantification of Picro Sirius Red staining of heart tissues in Figure 2K. **(K, L)** Picro Sirius Red staining and quantification of interstitial fibrosis sin heart tissues of mice. Representative staining images are shown in Panel K [scale bar = 50 μm]. Quantification is shown in Panel L. All quantitative figures showing Mean ± SEM; n = 6; * = p < 0.05 and ** = p < 0.01 compared to WT-Sham.

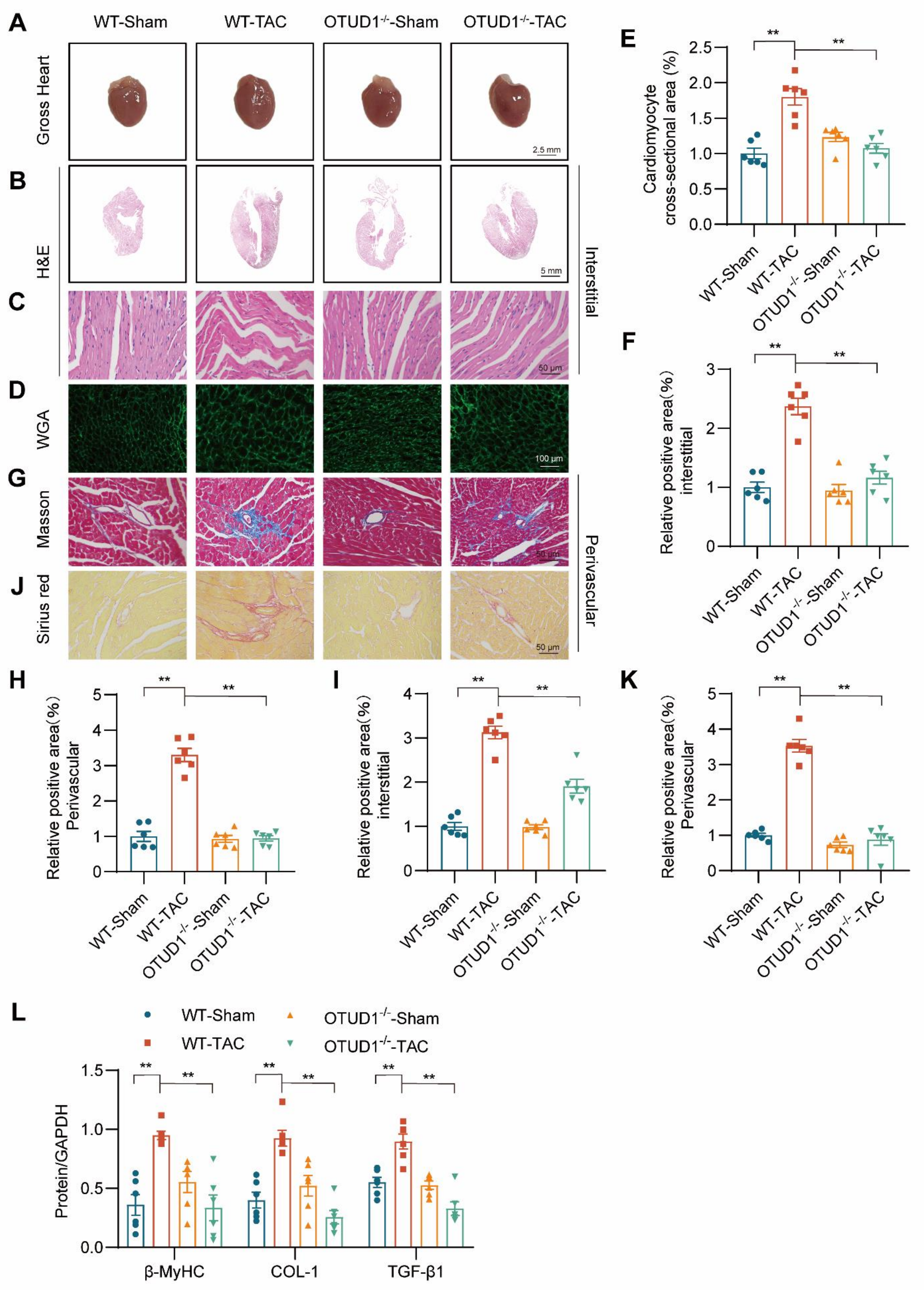
A
WT-Sham
WT-TAC
OTUD1-/--Sham
OTUD1-/--TAC
Gross Heart
2.5 mm
B
H&E
5 mm
Interstitial
C
50 µm
D
WGA
100 µm
G
Masson
50 µm
Perivascular
J
Sirius red
50 µm
E
Cardiomyocyte cross-sectional area (%)
F
Relative positive area(%) interstitial
H
Relative positive area(%) Perivascular
I
Relative positive area(%) interstitial
K
Relative positive area(%) Perivascular
L
WT-Sham
WT-TAC
OTUD1-/--Sham
OTUD1-/--TAC
Protein/GAPDH
β-MyHC
COL-1
TGF-β1

Figure S3: OTUD1 deficiency prevents TAC-induced myocardial hypertrophy and fibrosis. C57BL/6 wildtype (WT) and OTUD1 knockout mice were subjected to TAC. Tissues were harvested after 4 weeks and assessed. **(A)** Representative whole heart images [scale bar = 2.5 mm]. **(B, C)** Hematoxylin and eosin (H&E) staining of heart tissues from mice. Whole hearts (B) and high-power stained slices (C) are shown [scale bar = 5 mm (B) and 50 μm (C)]. **(D)** Cardiomyocyte size changes were detected by staining heart tissues with wheat germ agglutinin (WGA) [scale bar = 100 μm]. **(E)** Quantification of cardiomyocyte size, from WGA-stained heart tissues. **(F)** Quantification of interstitial fibrotic area (%) from Masson's trichrome-stained heart sections in Figure 3I. **(G, H)** Masson's Trichrome staining of heart tissues for perivascular fibrosis [scale bar = 50 μm]. Quantification of fibrotic area (%) is shown in Panel H. **(I)** Quantification of interstitial fibrotic area (%) from Picro Sirius Red staining in Figure 3J. **(J, K)** Interstitial fibrosis was detected in heart tissues by Picro Sirius Red staining (J) [scale bar = 50 μm]. Quantification of staining is shown in Panel K. (scale bar, 50 μm for Sirius Red staining). **(L)** Densitometric quantification of blots in Figure 3K. All quantitative figures showing Mean ± SEM; n = 6; * = $p < 0.05$ and ** = $p < 0.01$ compared to WT-sham.

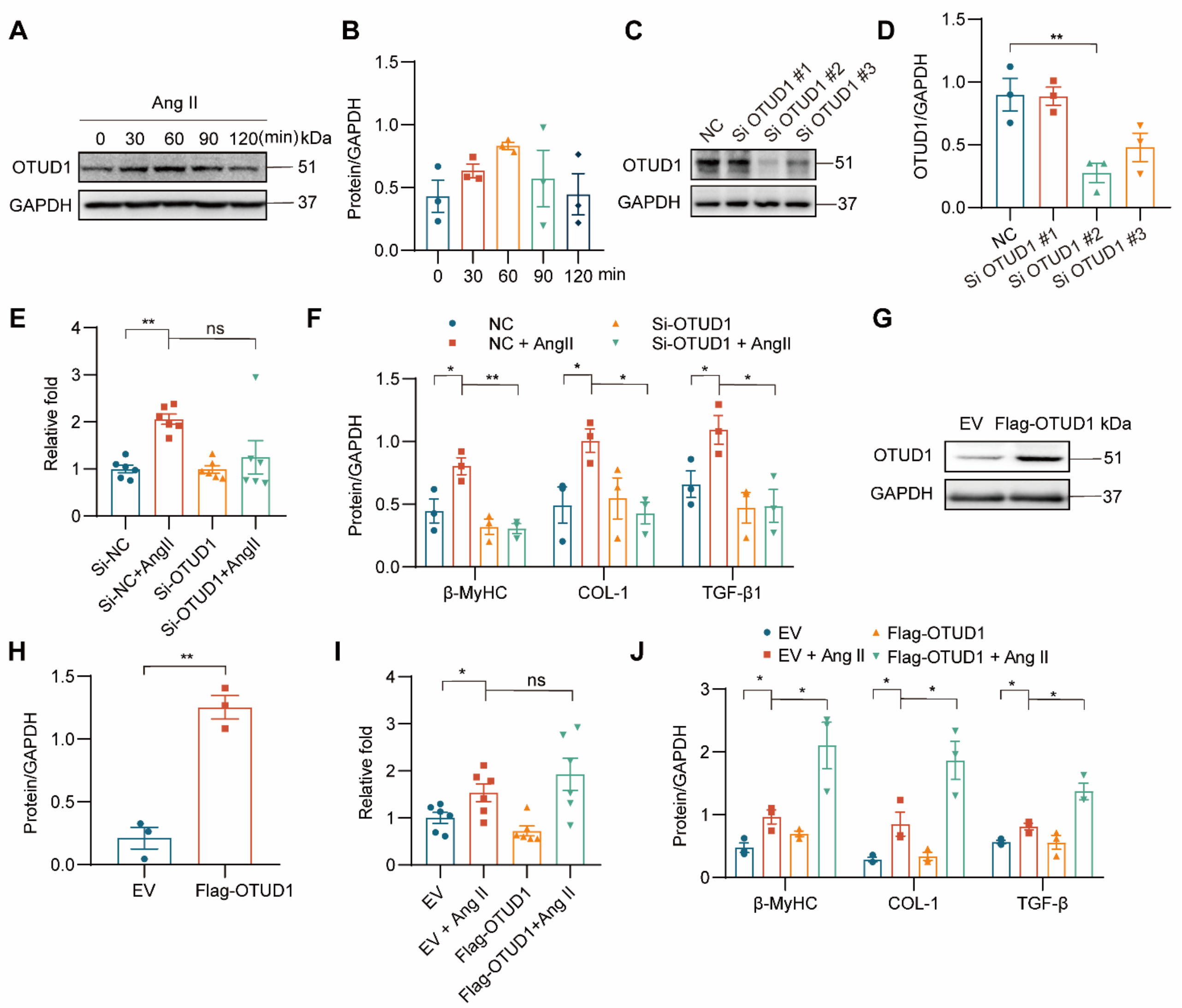

Figure S4: OTUD1 deficiency suppresses Ang II-induced cardiomyocyte hypertrophy and fibrotic responses *in vitro.*

(A) Representative western blots for OTUD1 protein in primary cardiomyocytes (neonatal rat ventricular cardiomyocytes; NRVMs) following Ang II exposure for different times. GAPDH was used as loading control. **(B)** Densitometric quantification of OTUD1 immunoblots in panel A. **(C, D)** OTUD1 gene silencing was performed in NRVMs by siRNA (si-OTUD1). Negative control (NC) included scrambled siRNA transfections. After 48 h, levels of OTUD1 proteins were detected by immunoblotting (C) and densitometric quantification (D). **(E)** Quantification of cell size changes in NRVMs in response to Ang II in Figure 4A. A minimum of 100 cells were measured from different visual fields of 3 samples per group. **(F)** Densitometric quantification of blots in Figure 4B. **(G, H)** OTUD1 was expressed in NVRMs using Flag-tagged OTUD1 vector. Control included empty vector (EV) transfections. After 48 h, levels of OTUD1 proteins were detected by immunoblotting (G) and densitometric quantification (H). **(I)** Quantification of cell size increase in NRVMs in response to Ang II in Figure 4E. A minimum of 100 cells were measured from different visual fields of 3 samples per group. **(J)** Densitometric quantification of blots in Figure 4F. All quantitative figures showing Mean ± SEM; n = 3 independent examinations; * = $p < 0.05$ and ** = $p < 0.01$.

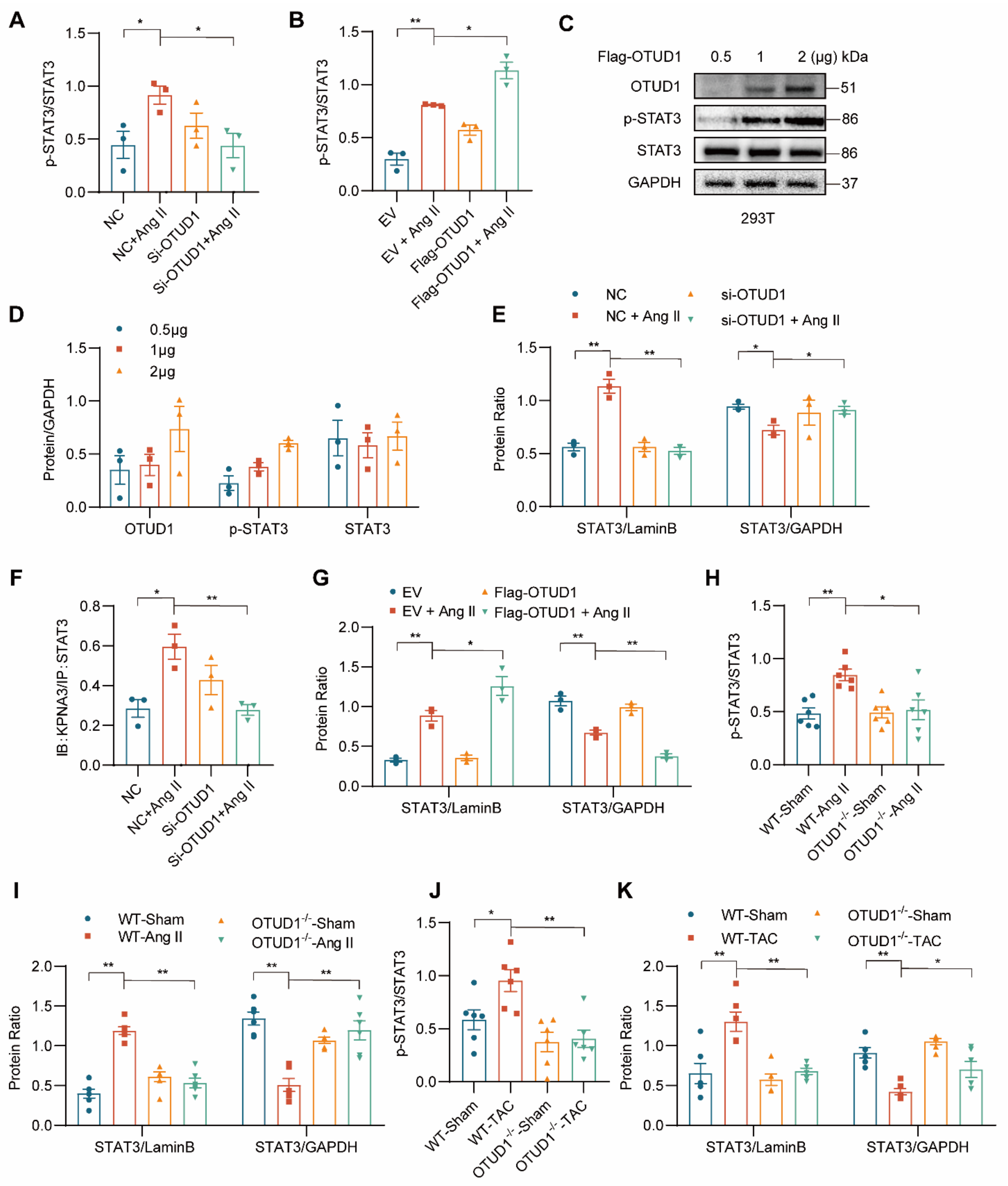

Figure S5: OTUD1 interacts directly with STAT3.

(A) Densitometric quantification of blots in Figure 5E. **(B)** Densitometric quantification of blots in Figure 5F. **(C, D)** HEK-293T were transfected with OTUD1 expressing vector. Levels of OTUD1 and (p-) STAT3 were detected by immunoblotting. Representative blots are shown in Panel C and densitometric quantitation is shown in D. **(E)** Densitometric quantification of blots in Figure 5G. **(F)** Densitometric quantification of blots in Figure 5H. **(G)** Densitometric

quantification of blots in Figure 5I. **(H-I)** Densitometric quantification of blots in Figures 5J and 5K. **(J**, **K)** Densitometric quantification of blots in Figures 5L and 5M. All quantitative figures showing Mean ± SEM; n = 3 independent examinations (Panels A-G) and n = 6 per group (Panels H-K); * = $p < 0.05$ and ** = $p < 0.01$.

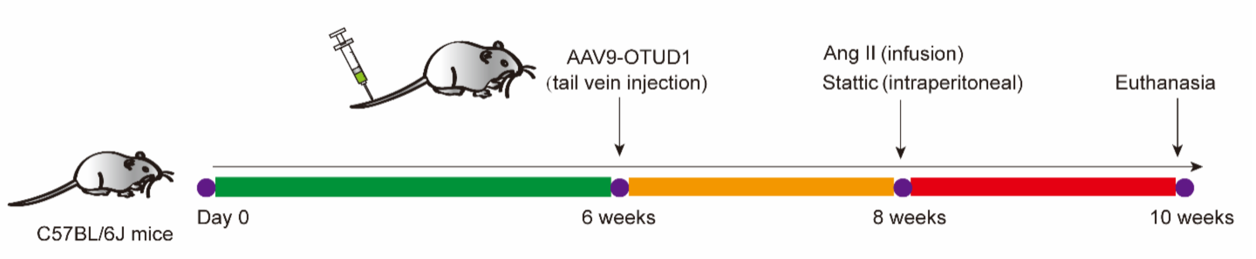

Figure S6: The flow chart of Ang II model with AAV9-OTUD1 injection and Stattic treatment.

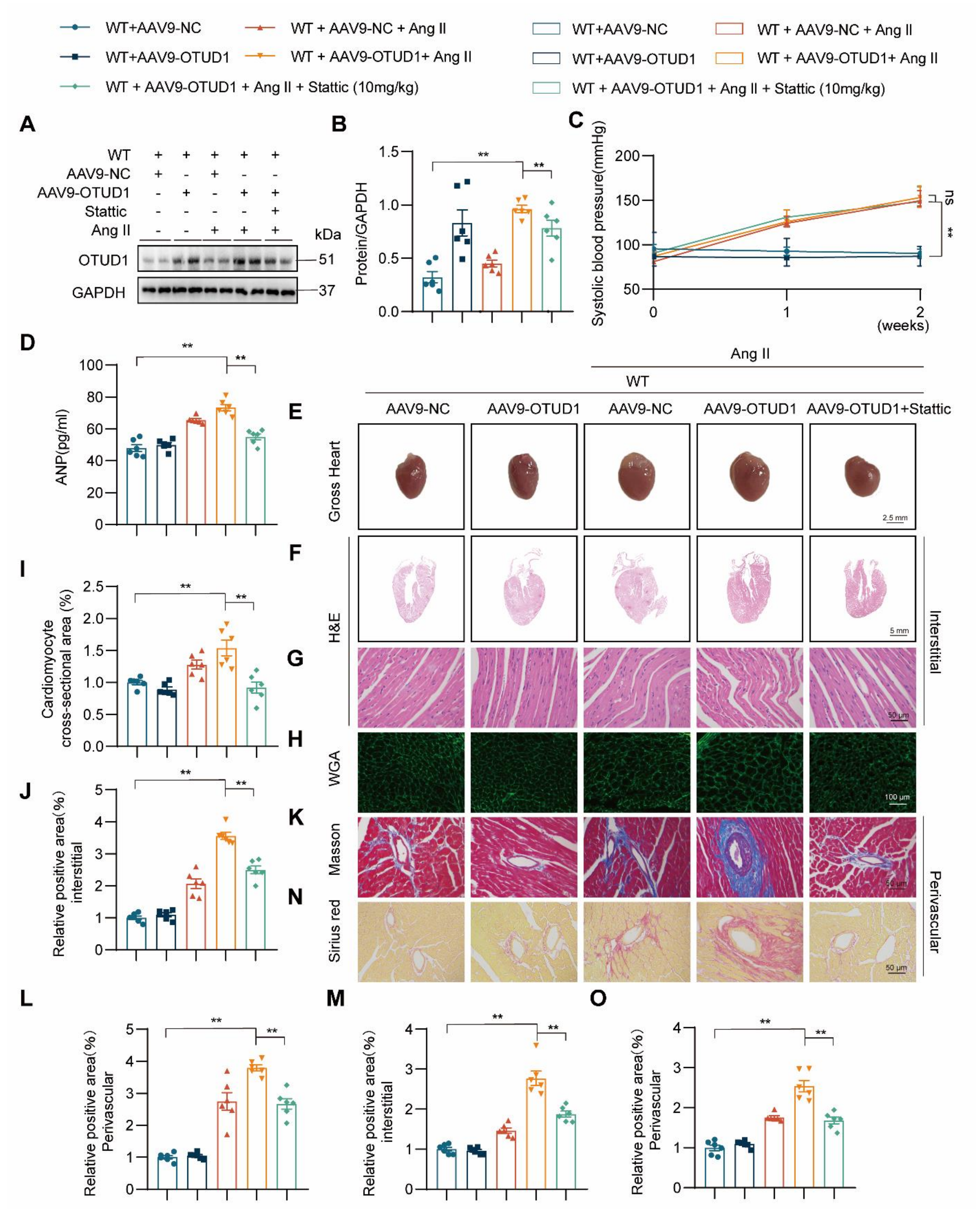

Figure S7: OTUD1 prevents Ang II-induced myocardial hypertrophy and fibrosis by regulating STAT3.

We infected the 6 weeks old wildtype C57BL/6 mice with adeno-associated virus serotype 9 (AAV9) that encodes OTUD1 (AAV9-OTUD1) and negative control (AAV9-NC). Mice were injected with AAV9 via tail vein (3×10^{11} viral particles/mouse) for 2 weeks. **(A**, **B)** OTUD1

protein levels in heart tissues were measured by immunoblotting. Representative blots are shown in Panel A and densitometric quantification is presented in Panel B. **(C)** Systolic blood pressure was determined by non-invasive tail-cuff on a weekly basis. **(D)** ANP protein levels were measured in serum samples obtained from mice after 2 weeks of Ang II infusion. **(E)** Representative whole heart images [scale bar = 2.5 mm]. **(F**, **G)** Hematoxylin and eosin (H&E) staining of heart tissues showing whole hearts (F) and high-power slices of tissues (G) [scale bar = 5 mm (F) and 50 μm (G)]. **(H**, **I)** Cardiomyocyte hypertrophy was measured by staining heart tissues with wheat germ agglutinin (WGA). Representative staining images are shown in Panel H [scale bar = 100 μm]. Cell size measurements are shown in Panel I. **(J)** Quantification of interstitial fibrotic area (%) from Masson's Trichrome staining of heart tissues in Figure 7I. **(K**, **L)** Masson's Trichrome staining was performed to assess fibrosis in heart tissues of mice. Representative staining images are shown in Panel K [scale bar = 50 μm]. Quantification of fibrotic area is shown in Panel L. **(M)** Quantification of interstitial fibrotic area (%) from Picro Sirius Red staining of heart tissues in Figure 7J. **(N**, **O)** Heart tissues were stained with Picro Sirius Red to detect interstitial fibrosis. Representative staining images are shown in Panel N [scale bar = 50 μm]. Quantification of staining is presented in Panel O. All quantitative figures showing Mean ± SEM; n = 6; ns = not significant; * = $p < 0.05$ and ** = $p < 0.01$.

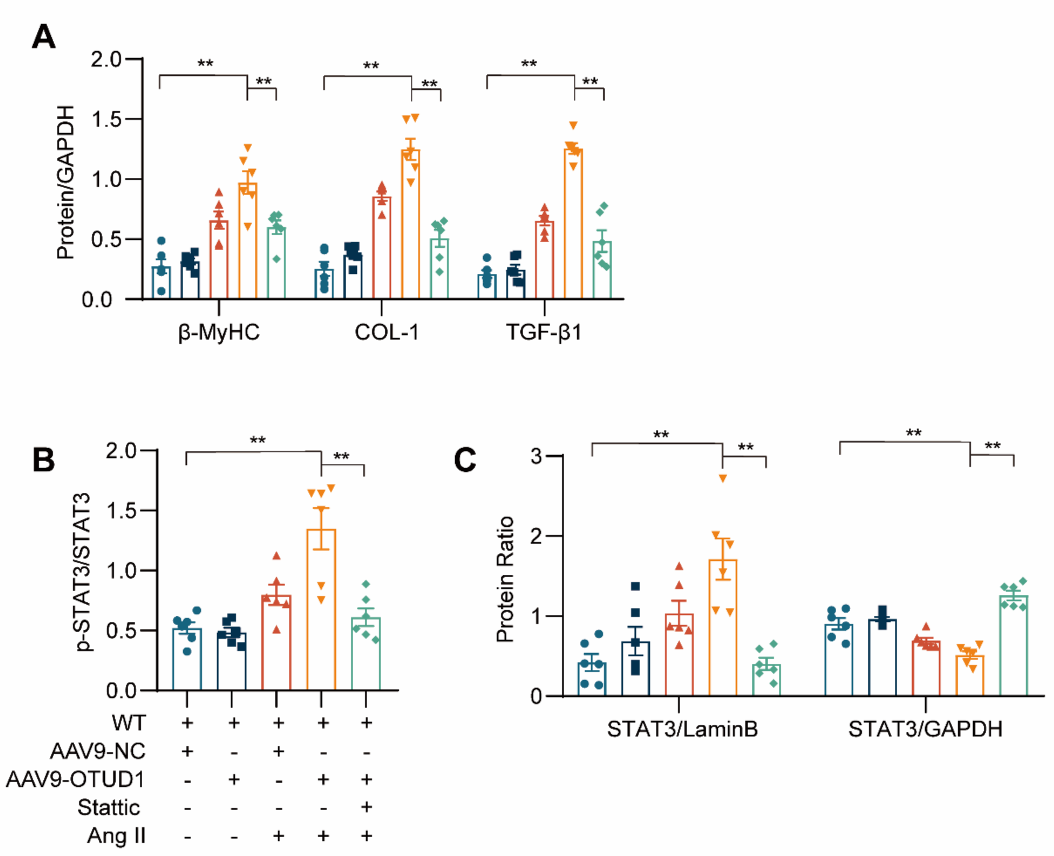

Figure S8: Fibrogenic factors and STAT3 activation in heart tissues of Ang II-challenged mice following OTUD1 expression.

(A) Densitometric quantification of blots in Figure 7K. **(B, C)** Densitometric quantification of blots in Figures 7N and 7O. All quantitative data is presented as Mean ± SEM; n = 6; * = $p < 0.05$ and ** = $p < 0.01$.
